# Infection of lung megakaryocytes and platelets by SARS-CoV-2 anticipate fatal COVID-19

**DOI:** 10.1007/s00018-022-04318-x

**Published:** 2022-06-16

**Authors:** Aiwei Zhu, Fernando Real, Claude Capron, Arielle R. Rosenberg, Aymeric Silvin, Garett Dunsmore, Jaja Zhu, Andréa Cottoignies-Callamarte, Jean-Marc Massé, Pierre Moine, Simon Bessis, Mathieu Godement, Guillaume Geri, Jean-Daniel Chiche, Silvana Valdebenito, Sandrine Belouzard, Jean Dubuisson, Geoffroy Lorin de la Grandmaison, Sylvie Chevret, Florent Ginhoux, Eliseo A. Eugenin, Djillali Annane, Elisabeth Cramer Bordé, Morgane Bomsel

**Affiliations:** 1 Laboratory Mucosal Entry of HIV and Mucosal Immunity, Institut Cochin, INSERM U1016, CNRS UMR8104, Université Paris Cité, 75014 Paris, France; 2 Service d’Hématologie Hôpital Ambroise Paré (AP-HP), Boulogne-Billancourt, France; 3 Hôpital Cochin, Service de Virologie, Hôpital Cochin (AP-HP), Paris, France; 4 INSERM U1015, Gustave Roussy Cancer Campus, Villejuif, France; 5 Electron Microscopy platform, Institut Cochin, INSERMU1016, CNRS UMR8104, Université Paris Cité, 75014 Paris, France; 6 FHU SEPSIS (Saclay and Paris Seine Nord Endeavour to PerSonalize Interventions for Sepsis), RHU RECORDS (Rapid rEcognition of CORticosteroiD Resistant Or Sensitive Sepsis), Department of Intensive Care, Hôpital Raymond Poincaré (APHP), Laboratory of Infection and Inflammation – U1173, School of Medicine Simone Veil, University Versailles Saint Quentin – University Paris Saclay, INSERM, Garches, France; 7 Service de Réanimation, Hôpital Ambroise Paré (AP-HP), Boulogne-Billancourt, France; 8 Université de Versailles-St Quentin en Yvelines, Versailles, France; 9 Service de Réanimation, Hôpital Cochin (AP-HP), Paris, France; 10 Department of Neuroscience and Cell Biology, University of Texas Medical Branch (UTMB), Galveston, TX 77553 USA; 11 Virologie moléculaire et cellulaire des coronavirus, Centre d’infection et d’immunité de Lille, Institut Pasteur de Lille, Université de Lille, CNRS, Inserm, CHRU, 59000 Lille, France; 12 Service d’Anatomie et cytologie pathologiques - Médecine legale, Hôpital Raymond Poincaré (AP-HP), Garches, France; 13 Service de Biostatistique, Hôpital Saint Louis (AP-HP), Paris, France; 14 Singapore Immunology Network (SIgN), Agency for Science, Technology and Research (A(∗)STAR), Biopolis, Singapore, Singapore; 15 Shanghai Institute of Immunology, Shanghai JiaoTong University School of Medicine, Shanghai, China; 16 Translational Immunology Institute, SingHealth Duke-NUS Academic Medical Centre, Singapore, Singapore

**Keywords:** Platelets, SARS-CoV-2, COVID-19, Megakaryocytes, Lung, Macrophages

## Abstract

SARS-CoV-2, although not being a circulatory virus, spread from the respiratory tract resulting in multiorgan failures and thrombotic complications, the hallmarks of fatal COVID-19. A convergent contributor could be platelets that beyond hemostatic functions can carry infectious viruses. Here, we profiled 52 patients with severe COVID-19 and demonstrated that circulating platelets of 19 out 20 non-survivor patients contain SARS-CoV-2 in robust correlation with fatal outcome. Platelets containing SARS-CoV-2 might originate from bone marrow and lung megakaryocytes (MKs), the platelet precursors, which were found infected by SARS-CoV-2 in COVID-19 autopsies. Accordingly, MKs undergoing shortened differentiation and expressing anti-viral IFITM1 and IFITM3 RNA as a sign of viral sensing were enriched in the circulation of deadly COVID-19. Infected MKs reach the lung concomitant with a specific MK-related cytokine storm rich in VEGF, PDGF and inflammatory molecules, anticipating fatal outcome. Lung macrophages capture SARS-CoV-2-containing platelets in vivo. The virus contained by platelets is infectious as capture of platelets carrying SARS-CoV-2 propagates infection to macrophages in vitro, in a process blocked by an anti-GPIIbIIIa drug. Altogether, platelets containing infectious SARS-CoV-2  alter COVID-19 pathogenesis and provide a powerful fatality marker. Clinical targeting of platelets might prevent viral spread, thrombus formation and exacerbated inflammation at once and increase survival in COVID-19.

**Supplementary Information:**

The online version contains supplementary material available at 10.1007/s00018-022-04318-x.

## Introduction

Since December 2019, the world has experienced an outbreak of coronavirus disease 2019 (COVID-19), caused by the severe acute respiratory syndrome coronavirus 2 (SARS-CoV-2). Although the epidemiological and clinical characteristics of patients with COVID-19 have been reported, biological risk factors for mortality are needed. Critical cardiovascular, as well as multifactorial thrombotic complications in patients with COVID-19 are frequent, even in individuals without a history of cardiovascular disease [1]. Furthermore, patients with severe COVID-19 admitted into intensive care unit (ICU) have increased cumulative thrombotic complications compared with patients not admitted to ICU (31% versus 1.3%) [2]. Microthrombotic events are especially frequent in the lung where MKs, the platelet precursors, are found to accumulate atypically in COVID-19 patients [3], suggesting abnormal behaviour [4]. However, anticoagulant treatment of COVID-19 patients is of limited efficacy, and any benefit may be patient-specific [5, 6]. Despite clinical evidence of a link between COVID-19 and haemostatic disorders, the underlying mechanisms of thrombosis remain uncertain.

The β-coronavirus SARS-CoV-2 is a single-strand RNA (+) enveloped virus [7]. The viral spike protein (S) is made of two subunits. The subunit S1 binds to its main receptors the surface expressed angiotensin-converting enzyme-2 (ACE-2) and is cleaved from the subunit S2 by the target cell serine protease TMPRSS2 [8], enabling  the virus to enter and infect target cells. Virus replication involves the production of double-stranded (positive (+)/negative (-) strands) complexes in the cytosol of the infected cells initiating viral component production. SARS-CoV-2 infected cells are found not only in the lung, but the virus is also widely found in other tissues [9]. The process of viral dissemination remains unknown. There is a lack of significant blood viremia, and only occasional detection of viral RNA due to rare blood cell infection [10–12]. Furthermore, no studies have so far demonstrated the presence of viral particles and their infectiousness. Therefore, the major route of SARS-CoV-2 dissemination is not blood, although the role of platelets as viral carrier has not been investigated.

Platelets have a critical role in hemostasis and thrombosis [13]. Their interaction with the subendothelium during viral infection results in platelet hyperactivity and in turn, arterial thrombus producing end-organ ischemia. In particular, the influenza virus can directly activate platelets [14, 15] with consequent uncontrolled coagulation cascade resulting in lung injury. Besides their role in hemostasis, platelets have immunological functions contributing to the immune response and inflammation [13, 16, 17]. Platelets can also harbor pathogens including viruses [18, 19] where some, such as Dengue virus, may even replicate [20]. Furthermore, HIV as well as Dengue and influenza virus can infect MKs [21], the cell producing platelets [21–23]. Accordingly, platelets can also shelter viruses such as HIV in vivo as we have recently shown [22], thereby participating in the propagation of the infection and altering the viral pathology. Platelets are hyperactivated in COVID-19 and transcriptomics found N and more often E genes associated with platelets in some patients, irrespective of disease severity [24, 25]. Whether MK infection occurs and replication-competent SARS-CoV-2 are contained in platelets with a possible role in COVID-19 thrombophilia, virus spread and patient outcome have not been addressed.

## Material and methods

### Patients and ethical statement

This non-interventional study was approved by the institutional review board of the ethical committee for research (CER) of the University of Paris-Saclay (CER-Paris-Saclay-2020-050) and conformed to the principles outlined in the Declaration of Helsinki. Accordingly, all participants were informed in writing about the study and allowed not to participate. We studied prospectively samples from 76 COVID-19 patients admitted at the Cochin (Paris, France), Ambroise Paré (Boulogne-Billancourt, France), and Raymond Poincaré (Garches, France) Hospitals between March and May 2020. All patients had COVID-19 diagnosis confirmed by SARS-CoV-2 RNA RT-qPCR in nasopharyngeal swabs at the hospital. For this study, blood (*n* = 52), bronchoalveolar lavages (BAL) (*n* = 19) and autopsy (*n* = 5) unpaired samples were obtained from severe COVID-19 patients.

Additional methods are detailed in supplementary material.

## Results

### Platelets from COVID-19 non-survivors harbor SARS-CoV-2

**Fig. 1:**
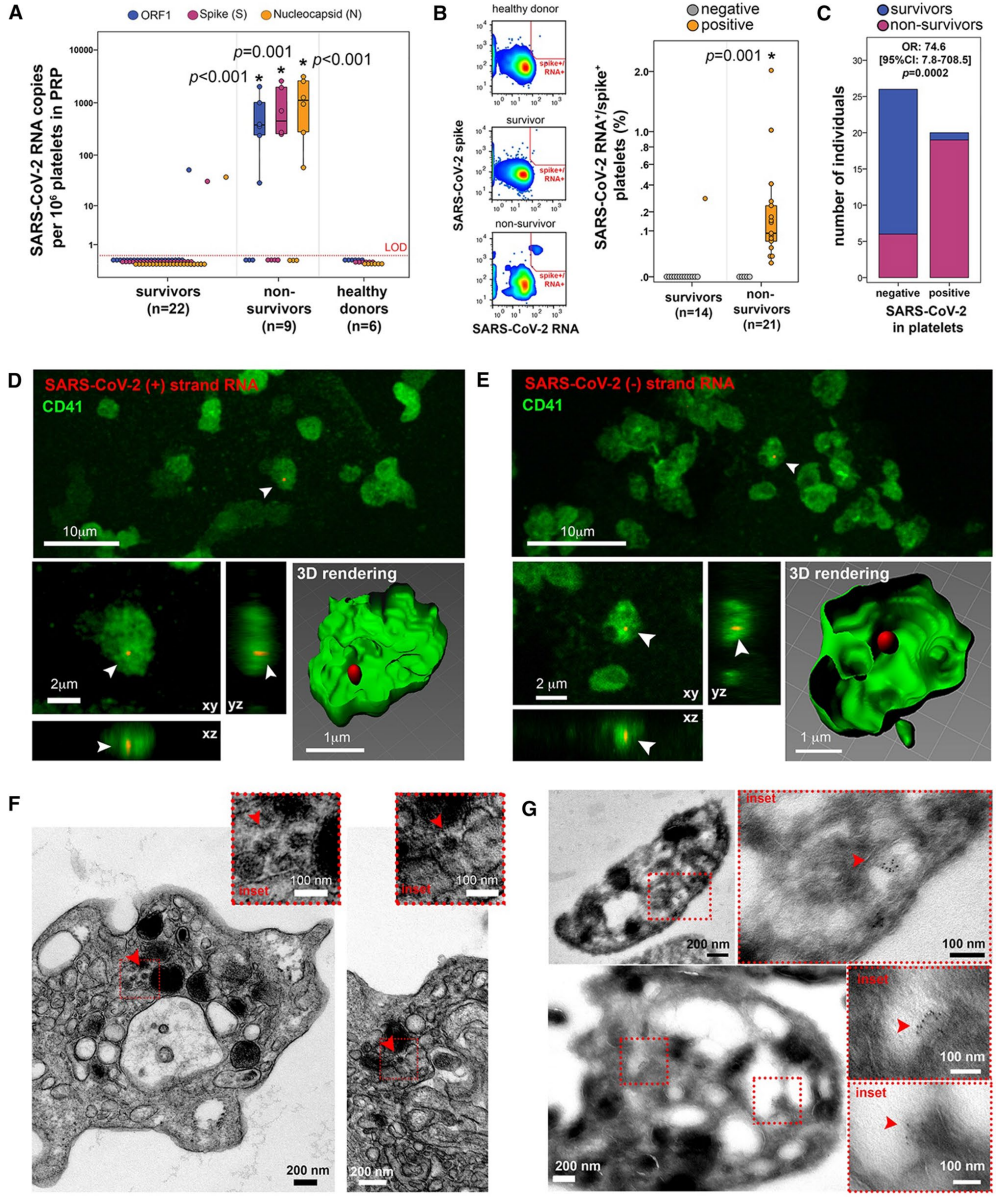
Platelets from non-survivor patients with COVID-19 harbor SARS-CoV-2. **A** Copies of SARS-CoV-2 ORF1 (blue), Spike (S, magenta) and Nucleocapsid (N, orange) RNA per million platelets detected by RT-qPCR, from COVID-19 survivors, COVID-19 non-survivors and healthy donor samples. Asterisk indicates statistical significance in the comparison between survivors and non-survivors per detected gene target (Kruskal–Wallis between the three groups). LOD = limit of detection. **B** Combined detection of SARS-CoV-2 spike protein and SARS-CoV-2 RNA by flow cytometry (FISH-flow). On the left, SARS-CoV-2 spike^+^/RNA^+^ detection gate (red) showing an example of healthy donor, COVID-19 survivor, and COVID-19 non-survivor. On the right, the percentage of SARS-CoV-2 spike^+^/RNA^+^ platelets among platelets from COVID-19 survivors and non-survivors, normalized by detected events in healthy donor samples. Samples were classified as negative (gray) or positive (orange) for the presence of SARS-CoV-2 in platelets. Asterisk indicates statistical significance in the comparison between survivors and non-survivors (Mann-Whitney between the two groups). **C** Number of COVID-19 survivors (blue) and non-survivors (magenta) among individuals tested for the presence of SARS-CoV-2 in platelets (negative or positive). OR: odds ratio. **D**-**E** Representative confocal microscopy images after CD41 (green) immunolabeling and SARS-CoV-2 RNA in situ hybridization (red) for SARS-CoV-2 (+) RNA (D) or SARS-CoV-2 (-) RNA (E) in platelet samples from a COVID-19 non-survivor. Images show low magnification (upper, bar = 10 μm), three-dimensional projections (xy, xz and yz, lower left, bar = 2 μm) and three-dimensional rendering (lower right, bar = 1 μm). Arrowheads indicate SARS-CoV-2 RNA, showing definite intracellular localization of the virus within the platelets. **F** Representative electron microscopy images of platelets with spherical crowned SARS-CoV-2 particles of 50-80 nm in diameter (arrowheads) located in the lumen of OCS in platelets of non-survivors (bars = 200 nm). Dotted line indicates area magnified as shown in the insets (bar = 100 nm). **G** Representative immunogold labeling with a polyclonal anti-spike antibody of non-survivor platelets otherwise tested positive for the presence of SARS-CoV-2 by FISH-Flow techniques (two examples, upper and lower images). Dotted squares point magnified regions where spike proteins are immunolocalized (red arrowhead). No spike immunolabeling was observed on platelet surface. Bar = 100, 200 or 500 nm

Platelet samples from randomly chosen individuals with confirmed severe COVID-19 diagnosis (*n* = 30) (Figure S1, Table 1, S1-2) were screened for SARS-CoV-2 by RT-qPCR (ORF, S and N genes). SARS-CoV-2 RNA was detected in 7 out of 30 patients. Strikingly, 6 out of the 7 platelet positive patients died within the week following sampling (mean days [95%CI]: 8.3 [5–14]), thus referred as non-survivors. Among the group of surviving patients (survivors), only 1 out of 24 platelet samples contained the viral RNA (Fig. 1A, mean [95% CI] RNA copies/million platelets in samples from non-survivors versus survivors, for ORF1 gene: 580 [160–1200] vs 2.2 [1.8–8.2], *p* < 0.001; for S gene: 840 [160–1600] vs 1.3 [1.1–5], *p* < 0.001; for N gene: 1200 [320–2000] vs 1.6 [1.3–5.6], *p* < 0.001). Using 11 of these samples and additional ones from 28 patients, we then evaluated if the RNA detected in platelets corresponded to full viral particles, using a flow cytometry technique for combined detection of SARS-CoV-2 RNA and spike proteins in platelets, referred to as FISH-Flow [22], which we now validated for SARS-CoV-2 (Figure S2 and Table S3). Detection of both viral RNA and protein was remarkably more frequent in platelets from non-survivors than from survivors (Fig. 1B, Figure S2, mean % [95%CI] of viral RNA^+^/spike^+^ platelets in samples from non-survivors versus survivors, 0.22 [0.06–0.42] vs 0.02 [0.01–0.07], *p* = 0.001).

**Table 1:** Patient characteristics according to the hospital outcome (Platelet samples)

Median [IQR]; *N*(%)	Survivors, *n* = 27	Non-survivors, *n* = 25	*p* value
Patients			
Age, years	61.6 [52.0;76.2]	72.7 [68.4;87.9]	**0.009**
Male sex	17 (63%)	16 (64%)	1.00
At least one comorbidity	22 (81%)	23 (92%)	0.42
Obesity	6 (19%)	2 (8%)	0.27
High Blood pressure	8 (30%)	10 (40%)	0.56
Cardiovascular disease	7 (26%)	8 (32%)	0.76
Diabetes	6 (22%)	10 (40%)	0.23
Active malignancy	5 (19%)	5 (20%)	1.00
Chronic renal failure	1 (4%)	2 (8%)	0.60
Chronic respiratory failure	4 (15%)	3 (12%)	1.00
At Hospital admission			
Days after first symptoms	10 [6.5;25.5]	8 [5;12.5]	0.23
Need of oxygen supply	2 (7%)	5 (20%)	0.24
O2 saturation, %	91 [88;98]	91.5 [82;95.75]	0.40
ICU admission	11 (41%)	12 (48%)	0.78
IGSII*	32 [30;39]	45 [34.25;57]	0.16
SOFA*	4 [3; 5]	4 [3.75; 8.75]	0.49
At sampling time			
Days after first symptoms	10 [6;26]	7 [5;12.5]	0.22
WBC count, Giga/L	7.4 [5.3;9.65]	9.2 [6.4;13.8]	0.26
Platelet count, Giga/L	238 [138.5; 334]	189 [160;255]	0.40
Plasma Fibrinogen, g/L	6.5 [5.175;7.525]	6.6 [4.9;8.1]	0.72
Plasma D-dimer, mg/L	4.4 [2.5;5.6]	3.5 [1.2;4.3]	0.091
Plasma vWF, μg/ml**	9.6 [7.448;14.35]	18.0 [12.56;27.33]	**0.015**
%vWF^+^ platelets**	5.9 [3.6;7.8]	14.5 [8.6;19.5]	**0.001**
SARS-CoV-2 in platelets	1 (4%)	19 (76%)	** < 0.0001**
At hospital discharge			
Days from first symptoms to discharge	25 [18;72]	15 [12.5;28]	**0.01**
Days from sampling to discharge	13 [10;44]	7 [5;10]	**0.001**

Bold indicates statistically significant *p* value (<0.05)

*Only available in those patients admitted to ICU (11 survivors and 12 deceased patients)

**Only measured in 16 survivors and 14 non-survivor patients

Taken RT-qPCR and FISH-Flow analyses together, SARS-CoV-2 virus was detected within circulating platelets in 20 patients out of 52, from which 19 died (Fig. 1C), in direct correlation with fatal outcome that persisted after adjustment for age (OR 63.4 [95% CI 6.6 to 610.1], *p* = 0.0003). Of note, the time from first symptoms to sampling was similar between survivors and non-survivors with mean value of 10 days [95% CI 6;26] and 7 [95% CI 5;12.5], respectively (*p* = 0.22), and thus did not introduce a bias in the analysis. The presence of SARS-CoV-2 in platelets was the strongest factor associated with fatal outcome with *p* < 0.0001 in a multivariate analysis based on all patients’ clinical data collected (Table 1).

In non-survivor patients, the virus resided inside platelets and was not associated to platelet surface: this was demonstrated by confocal microscopy after viral (+) and (-) strands RNA in situ hybridization using RNAscope technology [22] we validated (Figure S3A), coupled to immunostaining of the platelet marker CD41 (Fig. 1D–E and Figure S3B-C). In contrast, no viral signal was found in platelets from survivors. At the ultrastructural level, spherical crowned structures with a diameter of 50–80 nm and visible spikes at their periphery typical of SARS-CoV-2 [26–30] were detected inside non-survivor platelets (Fig. 1F and Figure S3D). These viral structures were found in subcompartments of the open canalicular system (OCS) [31] as well as in another type of compartment similar to the intra-platelet localization of dengue virus [32], whereas HIV [22], and Influenza viruses [33] are mainly found in the lumen of OCS. Immunolabeling of SARS-CoV-2 spike proteins on cryosections from these non-survivor platelets (Fig. 1G and Figure S3E) confirmed the SARS-CoV-2 nature of the viral spherical crowned images observed above (Fig. 1F and Figure S3D). Immunolabeled viruses were again exclusively detected within platelets, with no virus detected at the platelet surface. No such crowned viral structures nor immunolabeling were observed in survivor platelets. Platelets from non-survivors appeared also hyperactivated as indicated by the increase in frequency of platelets surface labeled for von Willebrand factor (vWF) in non-survivors versus survivors or healthy donors (Figure S4A-B) [34].

Although SARS-CoV-2 genes were scarcely found in blood [10, 11, 35], viral genes were more frequently detected in plasma (here in platelet-poor plasma (PPP)) from non-survivors than survivors (66% vs 25%) (Figure S4C). Furthermore, viral RNA copy number detected per million platelets in platelet-rich plasma (PRP) was not proportional to that detected per ml of PPP using RT-qPCR (Figure S4D), indicating that viral genes detected in the plasma are not the source of virus detected in platelets. In addition, platelets from healthy donor incubated with infectious SARS-CoV-2 were unable to internalize SARS-CoV-2 (Figure S4E-G), confirming that the source of SARS-CoV-2 in patient platelets was not endocytosis of virus possibly present in the plasma.

### In COVID-19 non-survivors, megakaryocytes are produced following a shortened megakaryopoiesis and express viral sensing genes

**Fig. 2:**
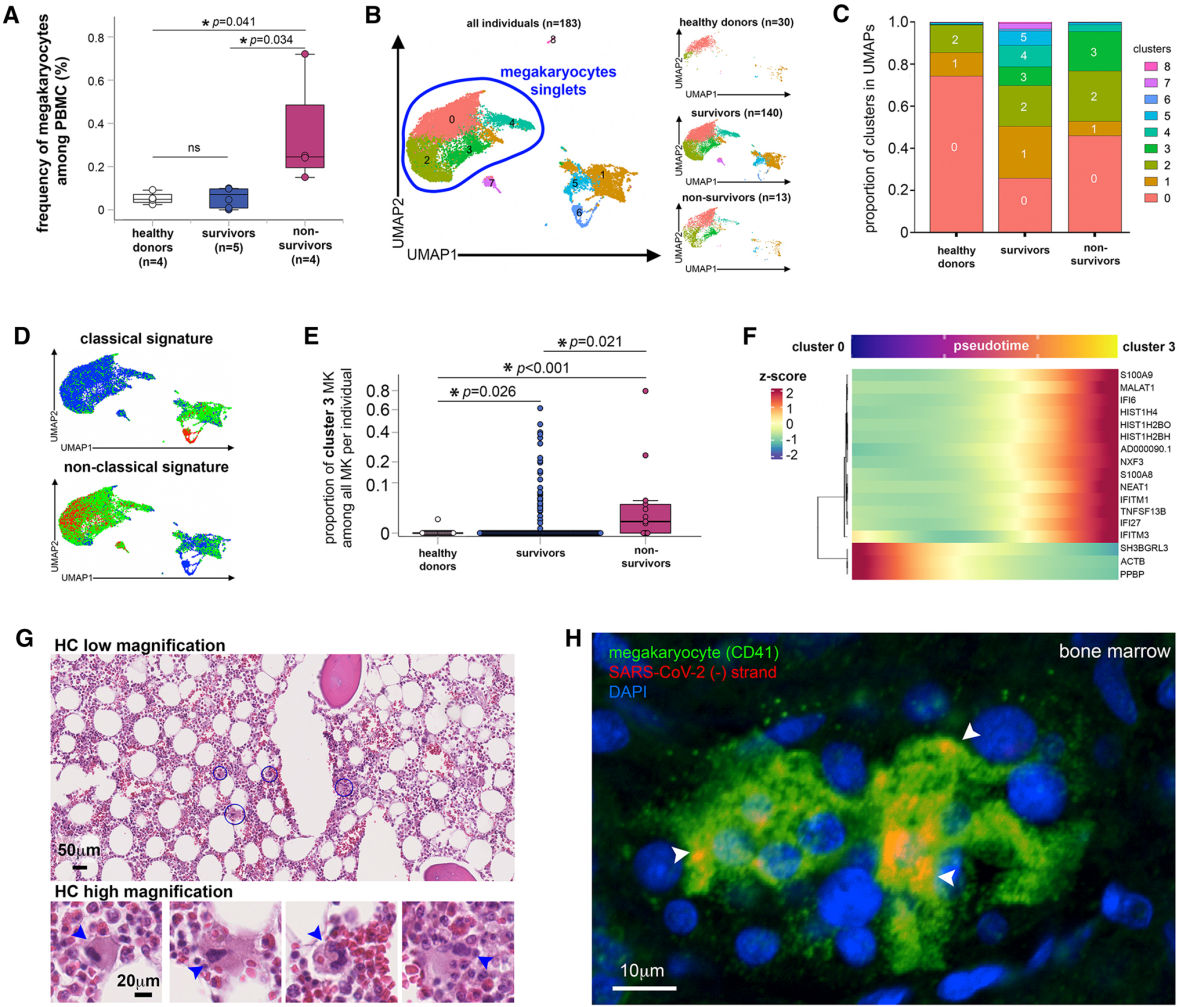
In COVID-19 non-survivors, MKs are infected and express viral sensing genes. **A** Frequency of MKs detected among PBMC from healthy donors and COVID-19 survivors and non-survivors as quantified by flow cytometry. Asterisks indicate statistical significance (Kruskal–Wallis test). **B**–**F** Transcriptional identity of MKs in COVID-19 patients by single-cell RNA sequencing reveals distinct phenotypes in non-survivor patients. **B** UMAP of single-cell transcriptomic data of MKs detected among PBMC from non-COVID-19 healthy donors (*n* = 30), COVID-19 survivors (n = 140) and COVID-19 non-survivors (*n* = 13). Unsupervised clustering detected 9 different clusters (0 to 8) of all cells analyzed. MK singlets are indicated by blue region. **C** Proportion of each cluster in healthy donors, COVID-19 survivors and non-survivors. **D** Scored gene signature expression of classical (upper) and non-classical (bottom) MK differentiation. **E** Fraction of cells from cluster 3 in comparison to all other clusters in individual patient samples categorized as healthy donors, COVID-19 survivors and non-survivors. **F** Heatmap of the genes that significantly change along pseudotime trajectory of MK development (*p* < 0.05 and Morans I score > 0.25). **G** Hematoxylin/eosin histology of bone marrow tissue obtained after COVID-19 non-survivor autopsy (low magnification (bar = 50 μm)) in which some MKs surrounded by blue circles are shown in high magnification insets (bar = 20 μm). Arrowheads indicate MKs. **H** Representative confocal microscopy images after CD41 (green) immunolabeling and replicative SARS-CoV-2 (-) RNA strand in situ hybridization (red) in bone marrow samples obtained from tissue autopsies of three different COVID-19 non-survivors (bar = 10 μm). Arrowheads indicate SARS-CoV-2 (-) RNA inside MKs

Alternative to virus endocytosis, platelets could have acquired SARS-CoV-2 in the bone marrow from their precursors, the megakaryocytes (MKs) previously infected during their maturation. This has been observed in Dengue and Influenza virus infection [21]. After entering the cava vein, bone marrow MKs reach the pulmonary circulation and then the lung [36]. These large MKs are filtrated in the pulmonary capillary bed producing platelets locally, but also releasing MKs with monolobed nucleus that are found later on in the peripheral circulation [37, 38]. Such monolobed MKs present in PBMCs were thus used as easily accessible surrogates for bone marrow MKs [4, 39]. Accordingly, monolobed MKs were detected at low frequencies in PBMCs from non-COVID-19 healthy individuals and COVID-19 survivors but their frequency strikingly increased in COVID-19 non-survivor samples (Fig. 2A and Figure S5A).

To further compare MK characteristics in COVID-19 non-survivors versus survivors and healthy controls, we integrated two PBMC single-cell RNA sequencing (scRNA-seq) data sets [4, 39] including healthy donor, COVID-19 survivor and non-survivor samples. Clustering analysis revealed that the MK transcriptome profile was more diverse in severe COVID-19 than in healthy controls (Fig. 2B, C and Figure S5B–D). Furthermore, genes associated with megakaryopoiesis in severe COVID-19 pointed to a shortened MK differentiation process. This shortened pathway is consistent with the non-classical pathway recently described that bypasses the MK-erythroid progenitor (MEP) step from the classical differentiation route [40], and associated with inflammatory conditions [41] (Fig. 2D and Figure S5E).

Strikingly, when compared with healthy donors and survivors, a unique MK profile identified as cluster 3, was enriched in non-survivors, both when cells were analyzed altogether (Fig. 2B, C) and by individuals (Fig. 2E). Resulting differentially expressed genes (DEG) in cluster 3 (Figure S6A–B) comprised platelet-secreted molecules upon activation such as PF4, inflammatory genes such as S100A8, a large set of IFN-stimulated genes (IFI6, IFI27, IFTIM3) that attest viral sensing, and genes driving megakaryopoiesis (PF4/CXCL4, MYL9, and histone associated genes). Accordingly, the pathways specifically associated with cluster 3 included not only rapid MK development and platelet functions, but also viral sensing by INF-stimulated genes and inflammation (Figure S6B-C). Cluster 3-enriched transcription factors such as JUND, GATA1, and RUNX1 were characteristic of megakaryopoiesis while NFKB1, RELA and STAT3 characterized an inflammatory immune response (Figure S6B). Inferred cluster 3 transcription factor targets were also enriched in IFN regulatory factors (Figure S6B). Among all trajectories rooted on cluster 0 (Figure S6D) that are predominant in MKs from healthy donors (Fig. 2B, C), the one transitioning MKs from cluster 0 to 3 (Figure S6D) confirmed not only the significant increase of histone-associated and inflammatory genes, but also that of antiviral IFN-stimulated genes (Fig. 2F).

### Infected bone marrow MKs in COVID-19 patients as a source of platelets containing SARS-CoV-2

Altogether, the scRNA-seq results indicated that MKs from COVID-19 non-survivors have matured faster than usual and have sensed the virus, thus being likely infected. In addition, MKs at an abnormally higher density were detected in bone marrow from COVID-19 versus non-COVID-19 autopsy cases (Table 2 and Fig. 2G, 49.89 ± 14.37 cells per mm^2^ versus 13 ± 3 cells per mm^2^, *p* = 0.0001), with an increased diameter size (103.36 ± 42.36 μm versus 30–100 μm). Furthermore, these bone marrow MKs recurrently contained replicative viral (-) RNA (Fig. 2H) with 14.99 ± 9.58% of the bone marrow MKs positive to (-) SARS-CoV-2 RNA (Figure S7A), establishing that MKs are infected in the bone marrow in COVID-19 non-survivors and constitute the likely source for producing platelets containing SARS-CoV-2.

**Table 2:** Patient characteristics according to the hospital outcome (autopsy samples)

**Patient**	**A1**	**A2**	**A3**	**A4**	**A5**
Sex	F	M	F	M	M
Age	82	51	59	51	71
ICU days	11	10	13	11	0
Days until demise	17	20	18	19	N.A.
BMI index	24.1	34.5	28.4	38.0	N.A.
Cardiovascular	No	AHT, AVB1	AHT	AHT	Low grade coronary illness
Pre-existing condition	N.A.	Small airways obstruction/ Leukemia	Asthma	Small airways obstruction/ Arthritis	Marked hepatic steatosis
Treatment	N.A.	AINS	Corticoids	N.A.	N.A.
Cause of death	Multivisceral failure	Pulmonary embolism	Pulmonary embolism	Massive pulmonary embolism	Major pulmonary edema
Lung sampling	Yes	Yes	Yes	Yes	Yes
Bone Marrow sampling	Yes	No	Yes	Yes	No

* F* female; *M* male; *AINS* non-steroidal anti-inflammatory drugs. *AHT* arterial hypertension; *AVB1* atrioventricular block level 1; *N.A.* not applicable

### Infected MKs are retained in the lung in COVID-19 non-survivors

**Fig. 3:**
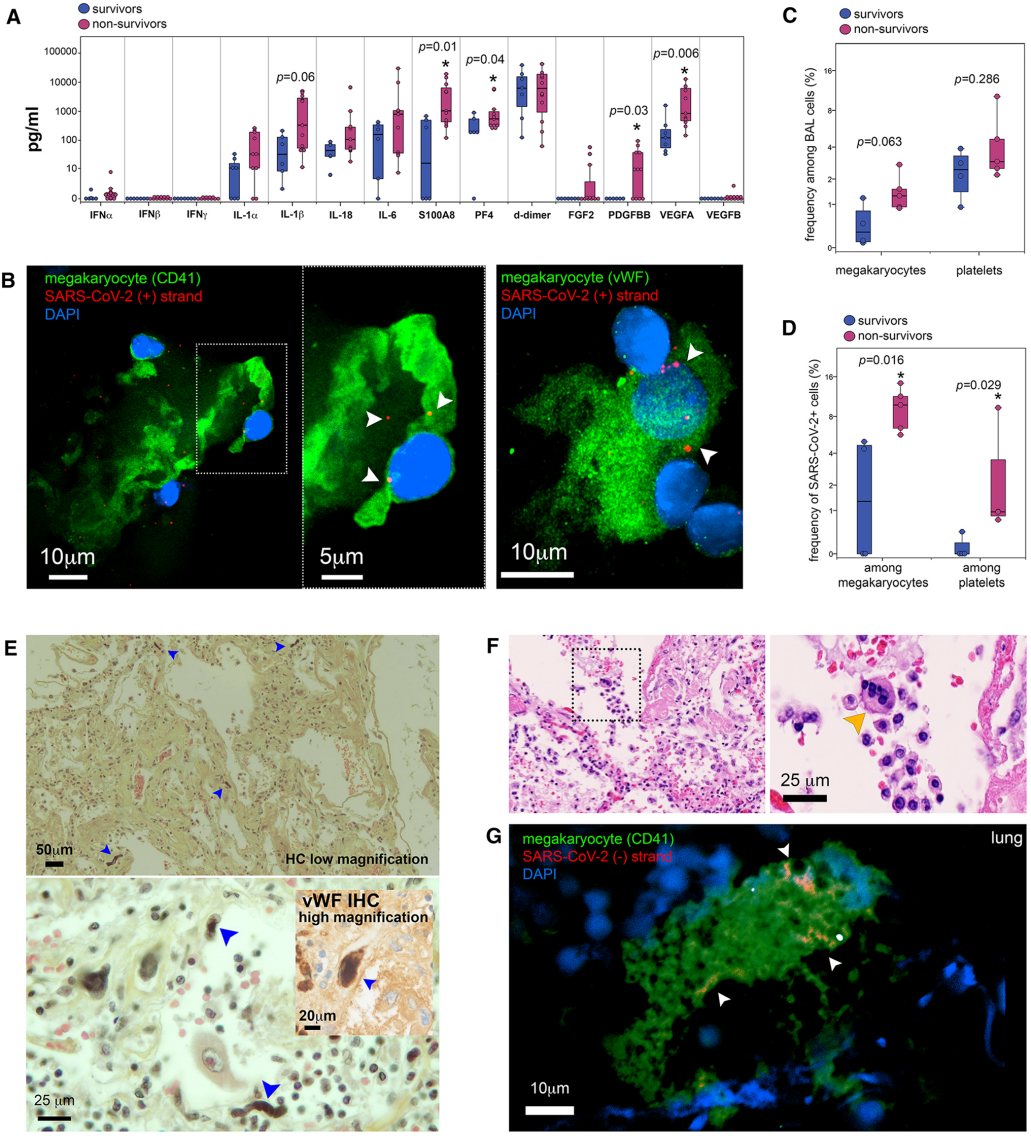
SARS-CoV-2 in platelets and MKs in lung from non-survivor COVID-19 patients. **A** Quantification of different cytokines/chemokines in bronchoalveolar lavage samples from COVID-19 survivors (blue) and non-survivors (magenta). Asterisk indicates statistical significance in the comparison between survivors and non-survivors (Mann–Whitney). **B** Representative confocal microscopy images after SARS-CoV-2 RNA in situ hybridization (red) for positive (+) RNA strand and immunolabeling of either CD41 (upper row) or vWF (lower row), both in green, in bronchoalveolar lavage samples from two different COVID-19 non-survivors (bar = 10 μm or 5 μm for the inset in upper image). Arrowheads indicate SARS-CoV-2 RNA inside MKs. **C** Frequency of MKs and platelets among the cell population detected in bronchoalveolar lavage samples from COVID-19 survivors (blue) and non-survivors (magenta) detected by flow cytometry. **D** Frequency of SARS-CoV-2^+^ MKs and platelets among the population of MKs and platelets detected in bronchoalveolar lavage samples from COVID-19 survivors (blue) and non-survivors (magenta) as quantified by flow cytometry. Asterisk indicates statistical significance in the comparison between survivors and non-survivors (Mann–Whitney). **E** Hematoxylin/eosin/saffron histology and vWF immunohistochemistry (lower inset) of lung tissue from COVID-19 autopsy, showing low (bar = 50 μm) and high magnification (bar = 20 and 25 μm) images. Blue arrowheads indicate MKs. **F** Hematoxylin/eosin histology of lung tissue in which some MKs indicated by dotted square region (left), resided to reside inside alveolar space (right) (bar = 25 μm). Orange arrowheads indicate MKs. **G** Representative confocal microscopy images after CD41 (green) immunolabeling and replicative SARS-CoV-2 (-) RNA strand in situ hybridization (red) in lung samples obtained from tissue autopsies of  five different COVID-19 non-survivors (bar = 10 μm). Arrowheads indicate SARS-CoV-2 (-) RNA inside MKs

Bronchoalveolar lavages (BAL) from severe COVID-19 patients provide an easily accessible fluid to probe the lung thrombotic and inflammatory environment during the progression of the disease. Thus, among a panel of factors implicated in hemostasis, inflammation and tissue repair analyzed, PF4/CXCL4 (platelet activation) and S100A8 (neutrophil and macrophage inflammatory activation) and VEGF-A and PDGF-BB (tissue repair and angiogenesis) were the only ones significantly increased in BAL from non-survivors compared to survivors (Table 3, Fig. 3A, PF4/CXCL4 mean pg/ml, 1487.8 [459.8–2798.7] vs 334.7 [111.9–630.6], *p* = 0.048; VEGF-A mean pg/ml, 3481.8 [1308.7–6077.6] vs 335 [68.9–826.4], *p* = 0.006; PDGF-BB mean pg/ml, 24 [6.7–43.5] vs 0, *p* = 0.037; and S100A8 mean pg/ml, 5004.7 [1481.4–8762.9] vs 242.2 [63.53–484.4], *p* = 0.01). This set of cytokines issued from or implicated with platelet/MK lineage which was enriched in BAL from non-survivor patients pointed to a virus-mediated MK impairment and retention in the lung, as already observed in the bone marrow in deadly COVID-19.

MKs appear specifically recruited into or retain within the lung in COVID-19 autopsy cases [3] together with endothelial damages [42, 43]. We, therefore, searched for MKs in BAL from patients with severe COVID-19 (Table 3). In line with the infection that we observed in bone marrow MKs, pulmonary MKs, identifiable through their large size and multilobed nucleus harboring cytoplasmic SARS-CoV-2 (+) RNA were detected in the BAL of COVID-19 patients irrespective of outcome, signing their viral infection (Fig. 3B and Figure S7B). When quantified by flow cytometry, a trend, although statistically non-significant, towards increase frequency of MKs and platelets among BAL cells from non-survivors compared to survivors was observed (Fig. 3C, Figure S7C). The frequencies of both infected spike^+^ MKs and virus-containing spike^+^ platelets in total MK and platelet populations were significantly increased respectively by 4- and 29-fold in BAL from non-survivors versus survivors (Fig. 3D, Figure S7C) (mean % spike^+^ MKs and platelets in BAL from non-survivors versus survivors, 9.5 [7.1–12.3] vs 2.3 [0.8–4.7], *p* = 0.016, and 2.9 [0.8–6.5] vs 0.1 [0.07–0.2], *p* = 0.029, respectively).

**Table 3:** Patient characteristics according to the hospital outcome (BAL samples)

Median [IQR]; *N*(%)	Survivors, *n* = 6	Non-survivors, *n* = 13	*p* value
Patients			
Age, years	54 [42;66]	69 [63;77]	**0.046**
Male sex	5 out of 6 (83%)	7 out of 13 (53%)	0.33
At least one comorbidity*	5 out of 6 (83%)	5 out of 11 (45%)	0.62
Obesity*	1 out of 6 (16%)	1 out of 11 (9%)	1.00
Cardiovascular disease*	2 out of 6 (33%)	4 out of 11 (36%)	1.00
Diabetes*	2 out of 6 (33%)	2 out of 11 (18%)	0.58
At hospital admission			
Days after first symptoms*	9 [6;9]	4.5 [3;7]	0.49
Days from Hospital admission to ICU admission*	1 [1;4.5]	1 [1;1.5]	0.83
ICU admission	6 out of 6 (100%)	13 out of 13 (100%)	–
Need of oxygen supply at ICU admission*	5 out of 6 (83%)	10 out of 12 (83%)	1.00
At Sampling time			
SARS-CoV-2 in BAL fluid	4 out 6 (66%)	6 out 13 (46%)	0.62
At hospital discharge			
Days from first symptoms to discharge*	46.5 [38.5;66]	41.5 [23;51.5]	0.57
Days from sampling to discharge*	21 [20;74]	25 [12;27]	0.89

Bold indicates statistically significant *p* value (<0.05)

*Only available indicated number of patients

The lack of statistically significant correlation between the frequency of spike^+^ platelets among total platelets recovered in BAL measured by flow cytometry and the viral load measured per ml of BAL by RT-qPCR (Figure S7D), further indicates that detection of virus in platelets is not a result of cell-free virus endocytosis from BAL.

Upon pathological examination of lung autopsy cases, a high density of MKs, identified by their size and the caterpillar appearance of their large nuclei, surrounded by fibrin webs, was detected by histochemistry (Table 2, Fig. 3E upper). Furthermore, capillary walls were highly damaged lacking the usual covering endothelial layer. MKs were not only found within the pulmonary vessels. Indeed, MKs also entered the alveoli (Fig. 3E lower) (Fig. 3E lower inset) and were found in the alveolar space as confirmed after specific vWF immunolabeling (Fig. 3F and Figure S7E) and in line with the unexpected presence of MKs in the BAL. When quantified in non-COVID-19 (*n* = 6) and COVID-19 (*n* = 3) lung autopsies as described [44, 45], the quantities of MKs increased by almost fourfold (from 5.78 ± 4.97 to 20.25 ± 6.99 cells per section. (5 mm^2^ and 5–7 μm thickness) in non-COVDI-19 vs COVID-19 autopsies: *p* = 0.0015), as well as their mean diameter (52.98 ± 21.98 μm in non-COVDI-19 vs 79.68 ± 19.45 μm in COVID-19 autopsies: *p* = 0.05) and nucleus size (26.6 ± 1.1 μm in non-COVDI-19 vs to 30.3 ± 1.2 μm in COVID-19 autopsies, *p* = 0.044, Figure S7F).

Furthermore, as observed in bone marrow and in BAL, lung tissue MKs were found actively infected harboring replicative viral (-) RNA (Fig. 3G). Indeed, 21.33 ± 12.1% (*n* = 3) of lung MKs were positive for SARS-CoV-2 RNA, and infected lung MKs were 2.5 ± 0.8(*n* = 3) times more frequently detected in alveoli than in blood vessels from lung tissues (Figure S7A). This suggests that platelets containing SARS-CoV-2 could also be produced in the lung of patients with deadly COVID-19.

Accordingly, a rough estimation of (-) SARS-CoV-2 positive platelet in tissue autopsies was obtained by measuring the Pearson’s correlation coefficients [46] for colocalization of SARS-CoV-2 RNA with CD41 after excluding the signal from large CD41 + polylobed MKs, and including CD41 signal from anucleated bodies only. As a result, correlation coefficients are significantly higher in both lung and bone marrow tissues from COVID-19 individuals (Pearson’s coefficient: 0.739 ± 0.252 and 0.747 ± 0.632, respectively) as compared with non-COVID-19 individuals (Pearson’s coefficient for lung and bone marrow tissues: 0.160 ± 0.093 and 0.122 ± 0.056, respectively) (*p* < 0.005, *n* = 3).

### Platelets containing SARS-CoV-2 are captured by pulmonary macrophages

**Fig. 4:**
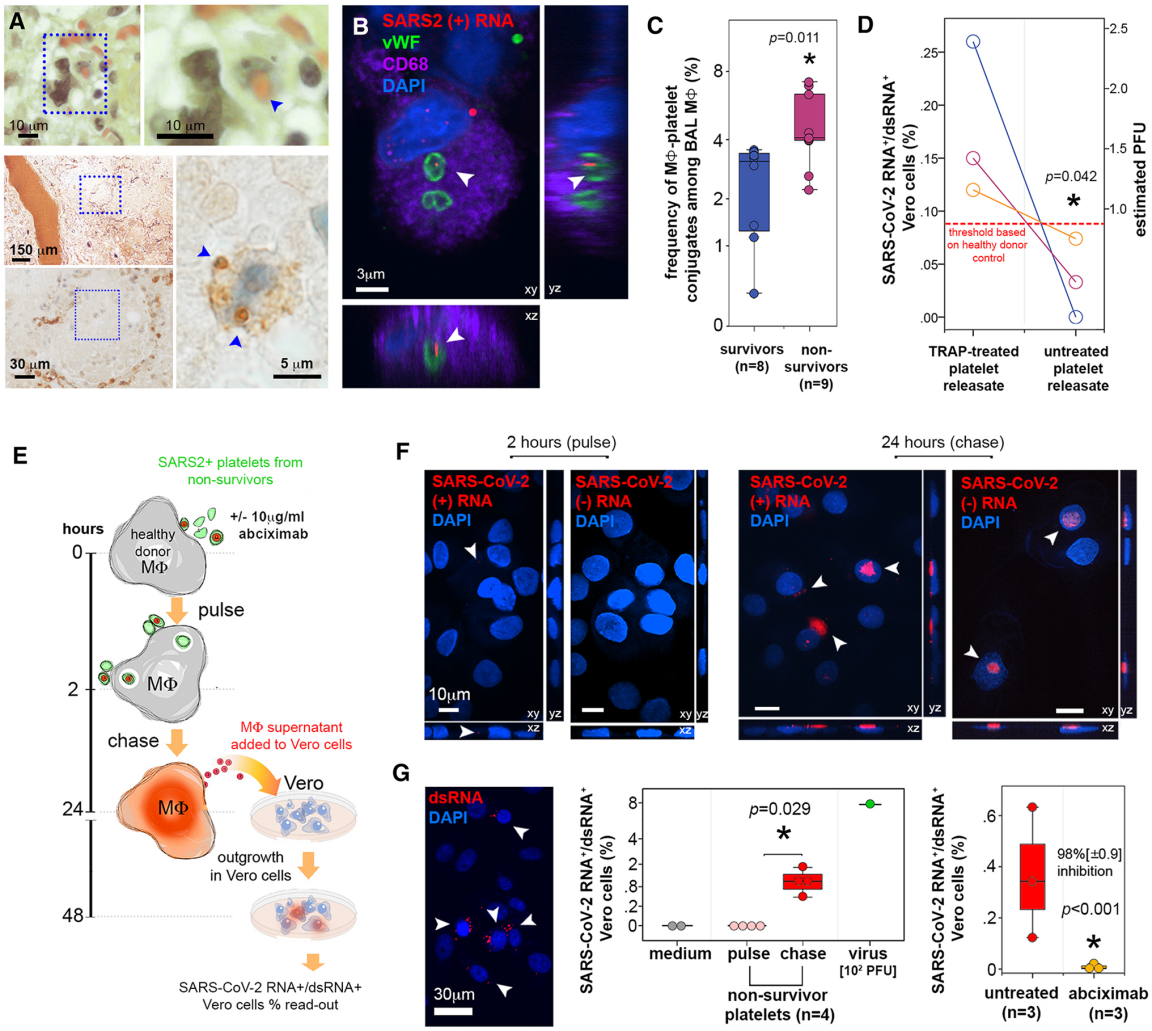
SARS-CoV-2 sheltered by platelets from non-survivor patients with COVID-19 is infectious to macrophages. **A** Upper row: Hematoxylin/eosin/saffron stain histology of representative lung tissue autopsy of COVID-19 patients showing a macrophage, indicated by dotted square region (left) in the process of phagocytosing a red blood cell as shown in higher magnification (right, blue arrowhead). Bar = 10 μm. Lower panel: Immunohistochemistry for vWF of alveoli from non-survivor lung autopsy (indicated by blue dotted square at low magnification, bar = 150 μm) where a macrophage (indicated by blue dotted square at middle magnification, bar = 30 μm) contained hemophagocytosed vWF^+^ platelets (blue arrowheads in high magnification image, bar = 5 μm). **B** Representative confocal microscopy images after vWF (green) and CD68 (purple) immunolabeling and SARS-CoV-2 RNA in situ hybridization (red) for positive (+) RNA strand in BAL samples from COVID-19 non-survivors. Images show three-dimensional projections (xy, xz and yz, bar = 3 μm). Arrowheads indicate SARS-CoV-2 RNA inside platelets engulfed by macrophages (representative of *n* = 3 different individuals). **C** Frequency of macrophage-platelet conjugates among macrophages in bronchoalveolar lavage samples from COVID-19 survivors (blue) and non-survivors (magenta) detected by flow cytometry. Asterisk indicates statistical significance in the comparison between survivors and non-survivors (Mann–Whitney). **D** Paired comparison of percentages of SARS-CoV-2 RNA^+^/dsRNA^+^ Vero cells treated with releasate from platelets treated or not with TRAP, from 3 different non-survivors. The detection threshold (dotted red line) was established with healthy donor platelets treated equally. The percentages were converted in PFU per million platelets using the standard curve we established. Mann–Whitney test. The estimated mean PFU per million platelets is shown in blue, with 95% confidence intervals. **E** Scheme of the experiments evaluating platelet-mediated SARS-CoV-2 transfer of infection to macrophages in vitro. SARS-CoV-2 -containing platelets from non-survivors interacted with macrophages in the presence or absence of abciximab (anti-GpIIbIIIa) for 2 h (pulse) followed by 24-h chase. At these time-points, macrophages harboring (+) and (-) SARS-CoV-2 RNA were enumerated by in situ hybridization and macrophage supernatants were collected and further evaluated for infectious virus content in reporter Vero cells. SARS-CoV-2 RNA^+^/dsRNA^+^ Vero cells were detected by in situ hybridization and quantified by FISH-flow. **F** Confocal microscopy images of SARS-CoV-2 RNA in situ hybridization (red) for positive (+) and negative (-) strand RNA in macrophages that interacted in vitro with platelets samples from COVID-19 non-survivors. Images were acquired after 2 h (pulse, left) or 24 h (chase, right) of interaction with platelets. Images show three-dimensional projections (xy, xz and yz, bar = 10 μm). Arrowheads indicate SARS-CoV-2 RNA. Macrophage nuclei are stained with DAPI (blue). **G** Outgrowth in Vero reporter cells of SARS-CoV-2 produced by macrophages after platelet-mediated infection. On the left, confocal microscopy image of double-strand RNA (dsRNA, red) in Vero cells cultivated with macrophage supernatants for 24 h. Arrowheads indicate infected Vero cells (bar = 30 μm), Vero cells nuclei are stained with DAPI (blue). On the middle graph, infected Vero cells were quantified by FISH-flow and expressed as % of SARS-CoV-2 RNA^+^/dsRNA^+^ Vero cells, comparing negative controls (medium, gray = non-infected Vero), positive controls (virus, green = primary SARS-CoV-2 obtained from patient bronchoalveolar lavage) and COVID-19 non-survivors platelets after pulse (2 h interaction with macrophages, light red) and chase (24 h after interaction with macrophages, red). Asterisk indicates statistical significance in the comparison between pulse and chase (Mann–Whitney). The right graph shows the outgrowth of SARS-CoV-2 in Vero cells incubated with macrophages that interacted with COVID-19 non-survivors’ platelets in the presence or not of abciximab (10 μg/ml) also quantified by FISH-Flow. Results are expressed as % of SARS-CoV-2 RNA^+^/dsRNA^+^ Vero cells in the two conditions. The % inhibition of Vero cell infection in the presence of abciximab is indicated, with asterisk corresponding to statistical significance in the comparison between the two groups (Student T-test)

The rupture of the endothelium from the lung capillaries and the epithelium covering the alveolar surface observed here in COVID-19 lung autopsies and as we reported [56] might let platelets penetrate the alveolar tissue and space. In turn macrophages could capture these platelets by hemophagocytosis [47–49], establishing a route to lung macrophage infection mediated by SARS-CoV-2-containing platelets and inflammatory modulation [50]. Indeed, hemophagocytosed platelets were detected in macrophages in the alveolar tissue and alveolar space of COVID-19 autopsies (Fig. 4A, Table 2). Furthermore, in BAL from additional severe COVID-19 patients, macrophages from non-survivors contained also platelets sheltering SARS-CoV-2 RNA (+) (Fig. 4B).

When analysed by flow cytometry, BAL from non-survivors had increased frequencies of platelet-macrophage conjugates than survivors (Fig. 4C and Figure S7C, mean frequency of platelet-macrophage conjugates among total macrophage in BAL from non-survivors vs survivors: 4.6 [3.5–5.8] vs 2.4 [1.5–3.3], respectively, *p* = 0.011). However, the frequency of macrophages (Figure S7G left) and spike^+^ lung macrophages (Figure S7G right) conjugated or not with platelets did not differ in BAL from non-survivors and survivors.

### SARS-CoV-2 contained in platelets of non-survivors is infectious

Whereas circulating SARS-CoV-2 free particles are not usually detected in infected patients [10], SARS-CoV-2 infected cells are found not only in the lung, but also at extrapulmonary sites in deadly COVID-19 [9]. We thus hypothesized that, in these patients, infection could be spread by platelets containing SARS-CoV-2 present in the circulation and at the pulmonary level.

We first investigated whether SARS-CoV-2 contained by platelets could be titrated on non-phagocytic Vero cells using the very sensitive FISH-flow assay we validated using different concentrations of SARS-CoV-2 (Figure S8).

To measure the titer of SARS-CoV-2 within platelets, we had first to force the virus to exit platelets from within, namely purging, by activating platelet with thrombin receptor activating peptide (TRAP) [51]. TRAP treatment induced virus release in the medium without platelet aggregation [52]. Platelets from COVID-19 non-survivors, with confirmed detection of SARS-CoV-2, were thus treated or not with TRAP, and centrifuged to collect platelet supernatants, referred to as releasates. Releasates were then titrated on Vero cells (Figure S9A). In paired samples of three different non-survivors, the percentage of SARS-CoV-2 RNA^+^/dsRNA^+^ Vero cells infected with TRAP-treated compared to untreated platelet releasates was statistically significantly higher (*p* = 0.042). Untreated platelet releasates fall below the limit of detection established using healthy donor platelet controls (Fig. 4D and Figure S9B). Vero cell infection was estimated in PFU using the PFU/FISH-flow standard curve (Figure S8D). From this calculation, the mean titer of SARS-CoV-2 virus sheltered in platelets and released by activating platelets was approximated to 35 [CI: 19–50] SARS-CoV-2 PFU per million platelets from COVID-19 non-survivors (*n* = 3) (Fig. 4D).

### Transfer of SARS-CoV-2 infection from platelets to macrophages is blocked by anti-platelet drug abciximab

Platelets are short-lived and are mainly eliminated following their capture by tissue macrophages [53]. Furthermore, lung macrophages express the main receptor for the virus and viral spike-priming protease necessary for infection (ACE2 and TMPRSS2) [54–56]. Lung macrophages are found infected in COVID-19 and support SARS-CoV-2 replication in vitro, as we and others have demonstrated recently [57–60]. They are thus a key cell in promoting viral spread [57–60] and the cytokine storm driving severe COVID-19 [50]. Primary macrophages derived from blood monocytes cultivated with M-CSF, interleukin (IL)-4 and IL-13 to mimic tissue macrophages [22, 61] and referred to here as MDM, expressed ACE2 and TMPRSS2, and supported SARS-CoV-2 replication with self-limiting release of infectious viruses (Figure S10A-D). As schematized in Fig. 4E, MDM were also incubated with platelets from non-survivors for 2 h (pulse), extensively washed and further cultivated for 24 h (chase). After the chase, both viral (+) and (-) strands RNA indicative of virus replication were detected in macrophage cytosol after in situ hybridization and confocal microscopy analysis (Fig. 4F right). In contrast, after the pulse, only the (+) RNA signal corresponding to the platelet inoculum was detected in MDM (Fig. 4F left). Viral RNA signal was also undetectable after incubation with platelets from survivors (not shown). Furthermore, infection of MDM mediated by platelet transfer of SARS-CoV-2 was productive. Indeed, when measured using Vero cells as reporter cells as in [62], replication-competent virus was detected in the 24 h supernatant of the MDM pulsed first with platelets containing virus for  2 h (Fig. 4G left image and middle graph). In contrast, no viral signal was detected after the pulse (Fig. 4G middle, % of SARS-CoV-2 RNA^+^/dsRNA^+^ Vero cells infected by the supernatant of MDM incubated with platelets samples from non-survivor after the pulse vs the chase, 0 vs 1.07% [0.6–1.5], *p* = 0.029). When titrated on Vero reporter cells, plasma deprived of platelets from survivors and non-survivors were not infectious (Figure S10E left). This experiment reveals the absence of infectious virus in the circulation despite viral gene detection. In contrast, plasma containing SARS-CoV-2 -positive platelets added to macrophages resulted in the production of infectious viral particles, titrated on Vero reporter cells (Figure S10E right).

Finally, transfer of infection from the SARS-CoV-2-containing platelets to MDM was blocked when platelets were pre-incubated with an anti-platelet GPIIbIIIa drug, abciximab, prior to MDM inoculation. In the presence of abciximab, the production of replication-competent virus by MDM after 24 h infection was reduced by 98 ± 0.9% (*p* < 0.001) (Fig. 4G right graph).

## Discussion

Platelet activation, thrombophilia and hypercoagulability have emerged as crucial pathological characteristics in severe COVID-19 that can lead to fatal outcome [1]. Hence, the frequency of thrombotic events in critical COVID-19 cases is particularly high, with increased frequency of venous and arterial thrombosis. These symptoms result in clinical complications including pulmonary embolism, ischemic stroke, and myocardial infarction [2, 6]. Increased platelet activation has been shown as a poor prognostic factor [34]. Furthermore, the presence of SARS-CoV-2 components in platelets has been reported in the context of COVID-19 platelet hyperactivation [24] [25].

The present study adds two novel platelet features that comfort the contribution of platelets to severe COVID-19. We not only demonstrate for the first time the presence of replication-competent functional virus in platelets but also correlate the detection of virus-containing platelets with patient outcome. In addition to contribution to life-threatening thrombotic disorders described in COVID-19 [1, 2], we now show that platelets can also harbor infectious SARS-CoV-2, detected as early as three weeks prior to death (median [IQR] days from sampling to death: 7 [5;10] [from 1 to 20]) (Table 1). Furthermore, in patients with COVID-19, the presence of infectious SARS-CoV-2 in platelets is a strong predictive marker of fatal outcome. The detection of SARS-CoV-2 in platelets by RT-qPCR or FISH-Flow, easily implementable as a routine analysis, could serve as a diagnostic useful tool to foresee as early as possible poor prognosis and take appropriate medical action.

The present results prompt the following three questions: how does the virus enter platelets, what is the possible causal effect of the presence of virus in platelets in COVID-19 pathology, and which type of treatment might prevent these effects.

As we found MKs, the platelet precursors, actively infected in the bone marrow and the lung in fatal forms of the disease, SARS-CoV-2 might likely associate with platelets during thrombopoiesis, in a process we and others demonstrated for HIV [22, 63], Dengue and Influenza virus [21, 23]. Alternatively, platelets could endocytose the virus in the circulation as they express SARS-CoV-2 receptors such as ACE2 [64] and DC-SIGN that participate in virus endocytosis, as we have shown in vitro [18, 65, 66]. It is, however, unlikely as free SARS-CoV-2 in platelet-poor plasma is not infectious and as platelets could not internalize SARS-CoV-2 in vitro.

Small MKs that reach the peripheral circulation after fragmentation from bone marrow and lung MKs [37, 38, 67], especially in inflammatory lung diseases [41], are present at low levels in PBMCs. Circulating small MKs can thus serve as surrogates in the analysis of human lung MKs, the access of which is challenging. Some of these circulating MKs harbor immunomodulatory functions in healthy donors [41, 68, 69]. Similarly, we found in the blood from COVID-19 non-survivors a significant increase of circulating MKs with a specific gene signature. These MKs carry type I IFN and inflammatory genes and show an upregulation of genes conditioned by NFKB1, characteristics of a response to viral infection including that to SARS-CoV-2 [70]. The expression of inflammatory genes typically involved in MK anti-viral response, such as IFITM1 and IFTIM3 [21], together with the non-classical MK differentiation pathway that we observed support the development of an emergency megakaryopoiesis in severe COVID-19, avoiding the MEP commitment [71]. Such shortened pathway might result in an increase in MK number and size, as we observed in bone marrow and lung tissues in COVID-19, compared to non-COVID-19 autopsies. Furthermore, we found in the lung of non-survivors an increase of CCL5, IL-1beta and IL6, cytokines inducing the production of thrombopoietin (TPO) that in turn speed megakaryopoiesis up, through the direct non-classical pathway [71]. Additionally, such increased CCL5, known to stimulate MK ploidy [71] and in turn speed megakaryopoiesis, could explain the increase in MK nucleus size we found in COVID-19 compared with non-COVID-19 lung autopsies.

Altogether, in line with the emergency myelopoiesis that we reported earlier [72], these results point to a MK response to pathogenic stimuli, i.e., infection by SARS-CoV-2 of MKs in the bone marrow, before entry in the circulation. Infected MKs would then migrate from bone marrow to the lung and produce platelets containing SARS-CoV-2 at both sites as we specifically detected in non-survivors. This finding are likely connected to COVID-19 fatal immunothrombosis.

However, no  appearance of thrombocytopenia or significant variation in platelets counts were detected in both survivors and non survivors when patient platelet counts of both patient sets were monitored during the following 15 days or until discharge for the non-survivors (Figure S1A). Of note, the increase in megakaryocyte numbers observed in autopsy tissues from COVID-19 versus non COVID-19 patients was not accompanied by an increase in platelet numbers during deadly COVID-19. This observation suggests either an inefficient thrombopoiesis due to shortened megakaryopoiesis; or alternatively, a retention of megakaryocytes in the lung of patients with an inefficient platelet production. Furthermore, as megakaryocytes are known to contain high amounts of growth factors and cytokines [73], they could in turn participate to a local cytokine storm.

MKs might also exert immunological functions by using the set of histone-associated genes that we found specifically increased in non-survivor inflammatory MKs. This may generate extra-nuclear histones that serve as damage-associated molecular patterns (DAMP) [74, 75], and participate in severing systemic inflammation and immunothrombosis in COVID-19 as it has been shown in sepsis [76, 77].

Infected MKs themselves would also participate to the cytokine storm by upregulation of interferon type I gene, as in other viral infections [21, 33, 78], and contribute to differential expression of virus-induced lung cytokines implicated in hemostasis and inflammation in COVID-19 non-survivors. Hence, PF4/CXCL4 is synthesized by MK and packaged within platelets alpha-granules during platelet production [13]. Increase in PF4/CXCL4 in BAL from non-survivors might reflect both MK destruction in pulmonary vessels and platelet activation, shown to occur in COVID-19 patients [34]. In turn, PF4/CXCL4 could intensify inflammation by polarizing lung tissue macrophages to the M4 inflammatory subtype [79]. and their secretion of  S100A8, thereby fueling inflammation [72, 79]. VEGF-A and PDGF-BB, secreted in particular by myeloid cells, such as MKs and macrophages [80], could represent stress responses to pulmonary endothelium damage [81, 82] that we observed in pulmonary autopsy tissues. VEGF-A binds to Neuropilin 1 (NRP1) on endothelial cells contributing to the integrity of the vessel wall and to the inhibition of platelet aggregation in blood vessels [80, 83]. SARS-CoV-2 spike protein that binds also to NRP1 [84, 85], may displace VEGF-A resulting in the increased levels of VEGF-A that we detected in COVID + BAL. Meanwhile, VEGF-A may facilitate small vessel thrombosis by inhibiting endothelial function both systemically and in the pulmonary circulation. In sum, the uncontrolled cytokine release may contribute to endothelial wall injuries [86]. These damages would allow entrance of MKs and platelets to the alveolar parenchyma as we observed in lung tissue autopsies and to the alveolar space as detected in BAL. This process appears specific to COVID-19, increased in non-survivors, although remaining limited in survivors.

Fully functional SARS-CoV-2 virus found within circulating platelets of non-survivors is infectious as in other viral infections [20, 22]. This indicates that SARS-CoV-2 viruses, protected by the platelet membrane from degradation by assault of antibodies and complement, might disseminate to other tissues. Virus would then propagate infection, contributing to rapid multiple organ failure [87, 88]. Accordingly, non-survivors in this study reach a fatal issue within 2 weeks after symptom onset (Table 1), when virus is still replicating [10]. We demonstrate that platelets harboring SARS-CoV-2 are capable to propagate infection to macrophages in vitro, a process abrogated by the anti-platelet GPIIbIIIa drug abciximab. We have already shown that blocking platelet GPIIbIIIa with abciximab fully prevents platelets internalization by macrophages [22], although by a mechanism that is not yet demonstrated. We can hypothesize that platelet GPIIbIIIa interacts with either macrophage CD40 [89] or the integrin Mac1 (αMβ2) in the presence of fibrinogen [90, 91] likely associated with platelets in our experimental conditions. This interaction would promote an outside-in GPIIbIIIa mechanism of activation that in turn triggers platelet uptake by macrophages [92] in a β2-mediated phagocytosis process [93]. Abciximab would impair such interactions and in turn, platelet-induced macrophage infection and/or possibly activation. This hypothesis remains to be verified.

In COVID-19 patients, lung tissue macrophages that we and other have found infected [57–60] may act as a Trojan horse: indeed, infected macrophges might transfer SARS-CoV-2 to nearby lung regions and in turn slowly propagating SARS-CoV-2 infection and spreading hyperinflammation across the lung [57–60], as witnessed by the increase in lung inflammatory cytokines we reported here. Upon phagocytosis by macrophages, SARS-CoV-2-containing platelets might transfer infection to macrophages that would produce IFN [94]. Incoming viral RNA could also signal Pattern Recognition Receptor (PRR) to enhance phagocytosis, increase oxidative burst and release of pro-inflammatory cytokines and chemokines, resulting in inflammation. This would promote an influx of monocytes HLA-DR low S100A8 + into the lung, sustaining inflammation and tissue damage [60]. Furthermore, platelet hyperactivation might be induced by virus or viral components within platelets. Activated platelets in turn would expose vWF (this study) and P-Selectin [34] triggering platelet aggregation, thereby contributing to lung inflammation as in influenza virus infection [95]. Based on our results, the anti-GPIIbIIIa drug may likely prevent these deleterious events. Therefore, therapeutic targeting of the platelet surface protein GPIIbIIIa could help blocking the process of viral spread in addition to its anti-thrombotic effect.

**Fig. 5:**
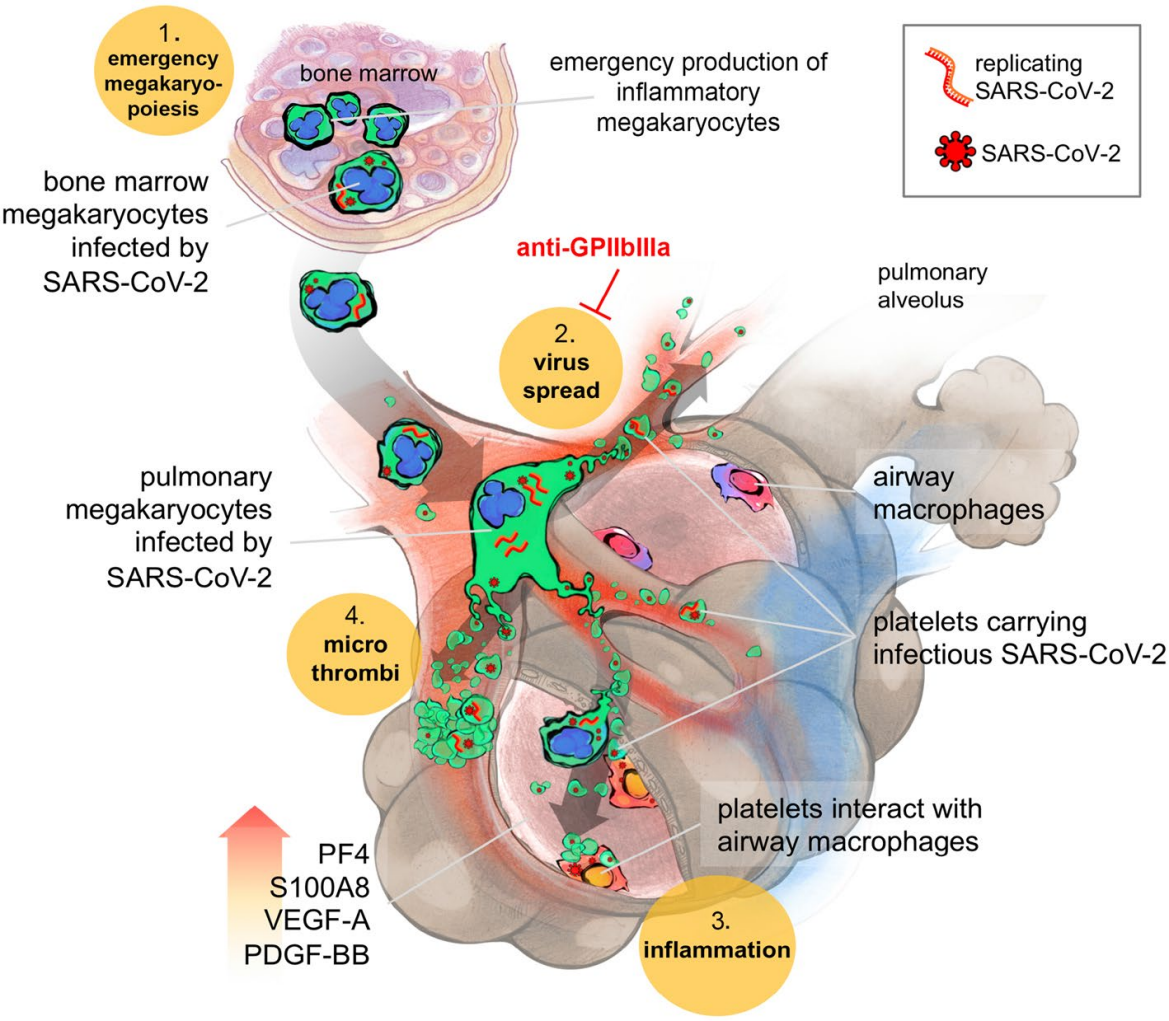
Scheme: Platelets harboring SARS-CoV-2 offer a convergent therapeutical target in severe COVID-19 with multiple manifestations. 1- SARS-CoV-2 favors emergency inflammatory megakaryopoiesis. SARS-CoV-2 infected MKs in the bone marrow (containing both viruses and replicating SARS-CoV-2 (-) RNA) migrate to the lungs where they contribute to thrombopoiesis and produce SARS-CoV-2-containing platelets in the pulmonary circulation. 2- These infectious platelets will then spread the virus and contribute to the systemic inflammatory component of severe COVID-19. As platelets sheltering SARS-CoV-2 are coated with von Willebrand Factor, indicating their highly activated status, they will also contribute to thrombus formation typical of COVID-19 complications. 3- Increase in lung VEGF-A and PDGF-BB participates to alveolar endothelial destruction and effraction allowing platelets carrying SARS-CoV-2 to reach and infect alveolar macrophages. 4- Increased lung PF4/CXCL4 released by platelets and S100A8 likely contribute to the maintenance of a highly inflammatory environment, macrophage activation, and cytokine storm. These four platelet-mediated components of severe COVID-19 suggest that targeting platelets, with the use of anti-platelet drugs like anti-GPIIbIIIa, might be an efficient strategy to block viral spread, thrombus formation and exacerbated inflammation at once, increasing the chance of survival

The following features we observed in non-survivors, namely increased phagocytosis of virus-containing platelets, endothelial cell wall injury, and the infection of MKs themselves, also occurring in other viral infection [21, 33, 78], may likely result in exacerbated macrophage-dependent cytokine production. As a result, thrombotic risk and complications [86], a hallmark of severe COVID-19, may increase. These deleterious effects of platelets harboring SARS-CoV-2 are summarized and schematized altogether in Fig. 5. Drugs targeting specifically the platelet receptor GPIIbIIIa may offer an alternative to scantily efficient anticoagulant treatment [6, 96]. Indeed, their direct and indirect anti-thrombotic effects, their prevention of macrophage infection and activation limiting their contribution to the cytokine storm, in addition to impairing the spread of infection to other tissue represent a valuable therapeutic recourse. Accordingly, a clinical trial using an anti-GPIIbIIIa drug in critically ill patients with COVID-19 has shown a beneficial effect on respiratory functions and clinical outcome [97]. In any case, the strategy of modifying platelet behavior needs careful consideration in the light of the results presented here. Indeed, we show that platelets from severe COVID-19 patients bear a dual prothrombotic effect: first via virally induced platelet activation and second, via decrease in endothelial functions caused by the virus blocking the effect of VEGF-A at the NRP1 receptor which may be considered as an appropriate therapeutical target.

The present study may likely apply to patients infected with various SARS-CoV-2 variants. Our study was performed in France during the first wave of COVID-19 between April and June 2020 with samples from patients infected by the ancestral Wuhan virus SARS-CoV-2 before the appearance of the following variants [98]. SARS-CoV-2 variants still use ACE2/TMPRSS2 for infection, although with different affinities [99]. Thus, variants might likely infect megakaryocytes resulting in the production of platelets containing SARS-CoV-2 that would likewise transfer infection to macrophages. We cannot rule out the possibility that infection by different variants would not affect megakaryocyte transcriptome and in turn the genes transferred to platelets during thrombopoiesis and downstream effects.

The scarce availability of platelet samples and the relative low frequency of virus-containing platelets, hindering detection by traditional techniques such as plaque assay is a limitation of this study. Hence, we applied the more sensitive single-cell FISH-flow technique [22] to approach infectious SARS-CoV-2. Importantly, the estimated quantity of infectious virus sheltered by platelets is non-negligible, being of 35 [CI 19–50] SARS-CoV-2 PFU per million platelets. Severe COVID-19 patients with fatal outcome appears to harbor 3.7–3.9 log_10_ platelet-associated SARS-CoV-2 PFU per ml of blood in the circulation. In addition, the contribution of the lung, the main site affected in severe COVID-19, has recently been estimated to half of the total platelet production with the lung producing 10 million platelets per hour [37]. This would translate into a lung production of around 2 log_10_ platelet-associated SARS-CoV-2 PFU/hour.

In sum, the presence of infectious SARS-CoV-2 in platelets combined with the intricate relationship between hemostasis, inflammation and the spread of infection has major consequences on COVID-19 pathogenesis and can turn out fatal. Anti-platelet drugs might be explored to develop anti-inflammatory coupled to anti-thrombotic treatment against severe SARS-CoV-2.

### Supplementary Information

Below is the link to the electronic supplementary material.

Supplementary file1 (DOC 11555 KB)

## Data Availability

Enquiries about data availability should be directed to the authors.
