# Supplementary material for: Infection of lung megakaryocytes and platelets by SARS-CoV-2 anticipate fatal COVID-19
Source: Cell Mol Life Sci. 2022 Jun 16;79(7):365. doi: 10.1007/s00018-022-04318-x (PMC9201269; doi:10.1007/s00018-022-04318-x)

**Infection of lung megakaryocytes and platelets by SARS-CoV-2 anticipate fatal COVID-19**

Aiwei Zhu et al.

**Supplementary Material and Methods**

**Sample collection**

***Platelet-rich Plasma (PRP):*** Peripheral blood samples were obtained in March and May 2020 from donors by venipuncture into plastic tubes containing anticoagulant ethylenediaminetetraacetic acid (EDTA). Samples were centrifuged for 10 minutes at 160g and 22°C for blood fractionation allowing for clear separation of PRP from whole blood. The upper two thirds of the PRP fraction were carefully collected and transferred to new plastic tubes. PRP fractions were frozen at -80ºC until use in the experiments. The quality and purity of samples were evaluated as described previously (1): more than 90% of platelets conserved membrane labeling of CD42b, a platelet labile glycoprotein marker (2), after thawing and the procedure does not increase the number of CD62P^+^ activated platelets (~50%)(Figure S1B). The number of leukocytes contaminating our PRP preparations is negligible with detection of 22.4 [CI: 14-32] leukocytes per ml of PRP corresponding to 1 leukocyte per 10^5^ platelets (Figure S1C). Platelet-poor Plasma (PPP) was obtained by centrifugation of PRP samples for 10 minutes at 1400g and collection of the supernatants. The clinical data of patients who have donated blood for PRP preparations (n=52) are summarized in Table 1.

***Autopsies:*** Lung and bone marrow were sampled on 5 autopsies from deceased individuals with confirmed SARS-CoV2 infection at the Forensic Medicine and Pathology, Versailles Saint-Quentin University, AP-HP - Raymond Poincaré Hospital, Garches, France. Hospital based autopsies were performed using all required protection. Bone marrow and lung samples correspond to discarded tissues from pathological analysis from COVID-19 infected areas. The clinical data of patients whose autopsy tissues were employed in this study (n=5) are summarized in Table 2.

***Bronchoalveolar lavage (BAL):*** Bronchoalveolar lavages were collected as described (3) and processed within 3 hours. BAL were passed through a 70-μm strainer, and collected in a 50 ml tube. After the centrifugation of 500 g for 10 min, fluid was collected and aliquoted at 1 ml and stored until use at -80 °C in biosafety level 3 (BSL3) laboratory. The BAL cell pellets were resuspended in 200 μl of the same individual BAL fluid, 20 μl of which were spotted and air-dried on SuperFrost Microscopic glass slides (Thermo Scientific) and kept in -80°C until use for *in situ* hybridization and confocal microscopy analyses. The BAL cells leftover was centrifuged again, resuspended in 10% Dimethyl sulfoxide (DMSO) in fetal calf serum (FCS) and stored in -80°C until use in flow cytometry analyses. BAL fluids without cells were aliquoted into 60 μl, inactivated in 56°C for 30 min in BSL3 facility and stored at -80°C for downstream use. The clinical data of patients who have donated BAL for PRP preparations (n=19) are summarized in Table 3.

**SARS-CoV-2 detection in platelets by FISH-flow**

SARS-CoV-2 viral particles in platelets were screened as described (1), using a flow cytometry single-cell approach which allows combined detection of both viral RNA (fluorescent *in situ* hybridization, FISH) and protein (immunolabelling) in platelets using samples at low amount. Cy5-tagged probe set for 4 SARS-CoV-2 gene targets (ORF1, RdRP within the ORF1 region, S and N, Table S3) were designed using Stellaris Probe Designer program (http://www.singlemoleculefish.com) based on a reference SARS-CoV-2 genome (NCBI: NC045512), and synthetized by LGC Biosearch Technologies. FISH was performed as described (1), using 12.5 nM of each probe per reaction. SARS-CoV-2 spike protein was immunolabelled before FISH, by indirect immunolabelling using a mouse IgG1 anti-SARS-CoV-2 spike antibody (2 μg/ml, Genetex, GTX632604) incubated for 30 min, room temperature, followed by incubation with 1:8000 (v/v) anti-mouse IgG1-AlexaFluor 488 antibody (Jackson, 115-545-205) in FISH-flow permeabilization buffer (1) for additional 30 min, room temperature. Platelets were immunostained using 1:50 (v/v) anti-CD41/CD61-PE antibody (Biolegend, 359806). A flow cytometry gating strategy for FISH-flow detection of SARS-CoV-2 in platelets was devised as described for HIV-1 detection in platelets (1). Results are expressed as the percentage of platelets double positive for SARS-CoV-2 RNA and spike protein (SARS-CoV-2 RNA^+^/spike^+^) among the CD41/CD61^+^ platelet population. The retrieved values were normalized by subtracting the maximum percentage obtained in healthy donor platelet samples evaluated in each experiment.

**Validation of FISH-flow using Vero cells**

SARS-CoV-2 FISH-flow method was validated using Vero cells infected with different concentrations of SARS-CoV-2 obtained from bronchoalveolar lavage of an infected individual. This virus had been previously titrated by the TCID_50_ standard method and the TCID_50_ titer converted to plaque forming units (PFU) (considering that 1.4 TCID50= 1 PFU) (4) (Figure S8A). Virus at different PFU was added to Vero cell cultures that were processed for FISH-flow quantification and the percentage of Vero cells positive for both SARS-CoV-2 FISH-flow (+) strand RNA probes and double-strand RNA (dsRNA) (SARS-CoV-2 RNA^+^/dsRNA^+^ Vero cells) (Figure S8B-C) statistically correlated with the viral input (PFU) (coefficient of determination R^2^ >0.9, p<0.01) allowing for appropriate conversion of FISH-flow data into PFU (Figure S8D). The dsRNA, indicative of SARS-CoV-2 replication (5) was detected by immunolabeling before FISH, by indirect immunolabelling using 1:500 (v/v) mouse IgG2a anti-dsRNA monoclonal antibody (J2, Scicons) (6) for 2 hours, followed by incubation with secondary anti-mouse IgG2a-FITC (Jackson, 115-095-206) for 1 hour at room temperature. The specificity of FISH-flow (+) RNA^+^ probes/ dsRNA^+^ data was further validated by searching for the presence of viral particles using triple labeling for spike protein, as described above but detected by a secondary antibody anti-mouse IgG1-BV711 (BD 742479), in addition to dsRNA and the SARS-CoV-2 FISH-flow (+) RNA probes. Results demonstrate that those double-positive FISH-flow (+) RNA^+^ probes/ dsRNA^+^ Vero cells do also contain viral proteins (Figure S8C). The specificity of SARS-CoV-2 RNA^+^/dsRNA^+^ Vero cells detection of replicating virus by FISH-flow was confirmed by treating Vero cells with the RNA dependent RNA polymerase inhibitor Remdesivir (MedChemExpress LLC) at 1, 5 or 10 μM for 30 minutes prior infection and maintained in Vero cell cultures throughout the time post-infection (Figure S2C).

**RT-qPCR**

Platelets were obtained from 500 μl of platelet-rich plasma (PRP) and processed for Reverse transcription and one-step quantitative polymerase chain reaction (RT-qPCR) as described (1). For BAL and PPP samples, total RNA was extracted from 150 μl of BAL fluid using NucleoSpin Dx Virus, Mini kit for CE certified purification of viral RNA/DNA (Macherey-Nagel) according to the manufacturer’s recommendations. RT-qPCR was performed from 50 μl of eluted RNA, using TaqMan RNA-to-C_T_ 1-step Kit (Applied Biosystems) and TaqMan 2019nCoV Assay Kit v1 (Thermo Scientific). Briefly, 4 targets are enrolled in our system. ORF1 gene (specifically the region encoding RNA-dependent RNA polymerase, RdRp), S gene (encoding Spike protein) and N gene (encoding nucleocapsid protein) aim at SARS-CoV-2, human RNase P RPPH1 gene runs in duplex with each SARS-CoV-2 assay, serving as an internal positive control. TaqMan 2019nCoV Control Kit v1 (Thermo Scientific) was used together to monitor assay-specific amplification. For each RT-qPCR reaction, a total volume of 20 μ comprises 5 μl of sample RNA, 10 μl of TaqMan PT-PCR Mix (2x), 0.5 μl of TaqMan RT Enzyme Mix (40x), 1 μl of TaqMan 2019nCoV assay and 1 μl of RNase P Assay. RT-qPCR was performed on a LightCycler 480 Instrument II (ROCHE). The reaction was carried out at 48 °C for 15 min, followed by an additional incubation at 95 °C for 10 min, followed by 40 cycles of 95 °C for 15 sec and 60 °C for 1 min. Results were analyzed using LightCycler 480 Software v1.5. For PRP samples, the number of SARS-CoV-2 RNA copies per target was normalized by the number of platelets in tested PRP volume as assessed by flow cytometry (CD41^+^ events), as expressed as copies per million platelets (1). Contamination of PRP by leukocytes was assessed by probing leukocyte transcript CD45 (PTPRC) (Hs04189704_m1, Thermo Scientific) using the TaqMan RNA-to-Ct 1-Step Kit protocol. The number of leukocytes was estimated from Cp values obtained from a standard curve using serial dilutions of RNA obtained from known amounts of human lymphoblast cell line CEM (ATCC-CRL2265). We have also analyzed by RT-qPCR the same ORF1, N and S genes in PBMC from the same donors whose platelets were positive for these targets using the same methodology. Although these patients were viremic and had virus associated with platelets, their PBMCs lack detectable levels of SARS-CoV-2 RNA. This is in agreement with the literature reporting that PBMCs are not infected or do not associate with viral components at levels detectable by the techniques we are employing (7).

The percentages of SARS-CoV-2^+^ platelets quantified in the same PRP samples by both RT-qPCR and FISH-flow show statistically significant correlation (Pearson correlation coefficient of 0.76, p=0.044) (Figure S2A), assuming that 1 RNA copy equals to one virus in RT-qPCR data (0.058% [CI: 0.016-0.12], 0.084% [CI: 0.016-0.16], and 0.12% [CI: 0.032-0.2], of total platelets contain SARS-CoV-2 RNA for ORF1, spike (S) and nucleocapsid protein (N) genes respectively). When compared, the percentages of SARS-CoV-2^+^ platelets by these two different techniques do not differ statistically (Figure S2B) and thus the two techniques can be used equally.

**SARS-CoV-2 detection in platelets by *in situ* hybridization and immunofluorescence**

To assess the localization of SARS-CoV-2 in platelets from PRP, 20 μl of PRP were fixed in suspension of 10% Neutral buffered formalin (NBF, pH=7.0). Fixed platelets were washed and resuspended in Phosphate buffered saline (PBS), and were air-dried as a 0.3 cm^2^ spots on SuperFrost slides coated with Cell-Tak (Corning). To assess the localization of SARS-CoV-2 in platelets from BAL, frozen air-dried BAL cell slides were taken out of -80°C and fixed immediately in 10% NBF for 30 min at room temperature. The glass slides with samples were processed for RNAscope in situ hybridization as described (8, 9), adjusting the time for RNAscope Target Retrieval Reagent treatment (322000, ACD) to 30 min and the time for RNAscope Protease Plus Reagent (322380, ACD) treatment to 5 min. Sample were incubated with RNAscope Probe-V-nCoV2019-S (848561, ACD) or RNAScope Probe-V-nCoV2019-S-sense (845701, ACD) for 2 hours at 40°C. Then, specific signals were amplified using RNAScope Multiplex Fluorescent V2 Assay Kit (323100, ACD) from step 1 to step 3 and further magnified by tyramide signal amplification (TSA), visualized by fluorescent dye Opal 570 (1:1500 v/v in TSA buffer, Akoya Biosciences). Samples were immunolabelled thereafter by direct immunofluorescence using 1:20 v/v anti-CD41 antibody coupled to APC (Biolegend, 303710), and by indirect immunofluorescence using 20 μg/ml rabbit polyclonal anti-CD41 (Thermo Scientific, PA5-96386), 10 μg/ml rabbit polyclonal anti-Von Willebrand Factor (vWF, abcam, ab6994) and 10 μg/ml mouse IgG2b anti-CD68 (R&D, MAB20401), followed by the secondary antibodies donkey anti-rabbit IgG coupled to FITC or Cy5 (Jackson, 711-096-152 and 711-177-003), and anti-mouse IgG2b coupled to Cy5 (Jackson, 115-175-207). Nuclei were labelled by 4',6-diamidino-2-phenylindole (DAPI, Sigma-Aldrich) for 10 min and slides were mounted using Ibidi Mounting Medium (ibidi, GmbH). Confocal images were acquired using an IXplore Spin Confocal Imaging Microscope System (Olympus) and processed by Imaris v9.0.2 Software (Oxford Instruments). The image processing includes fluorescence signal thresholding for the observation of RNAscope specific signal (using samples from COVID-19 negative individuals as signal threshold for RNAscope probe specificity), three-dimensional projections in xy, xz and yz sections and segmentation with three-dimensional rendering to ascertain virus localization (9).

**Titration of virus contained in platelets using platelet releasates**

To titrate the virus contained in platelets, we had to force the virus to exit platelets from within, namely purging it. A protocol of freeze-and-thaw cycles employed to purge Dengue virus from platelets (10) was inefficient for SARS-CoV-2. We thus performed a mild platelet activation with thrombin receptor activating peptide (TRAP) (11) that promote the release of platelet content without platelet aggregation (12). Accordingly, 200 μl of PRP from COVID-19 non-survivor individuals, containing SARS-CoV-2^+^ platelets as confirmed by either PCR or FISH-flow, were washed 2 times in 200 μl of Tyrode’s buffer (pH=7.4, NaCl 134mM, NaHCO_3_ 12mM, KCl 2.9 mM, Na_2_HPO_4_ 0.34 mM, MgCl_2_ 1 mM, HEPES 10mM) by centrifugation at 400 g, 10 min at room temperature. Platelet were then treated or not with TRAP (5μM in Opti-MEM I (Gibco, 11058-021)) for 10 minutes at 37°C, followed by centrifugation at 1100g to discard platelet pellets and to collect platelet supernatants referred to as releasates. Releasates were added to 10^5^ Vero cells/well in 48-wells plate cultures in the presence of TPCK for 24 hours prior to detection of SARS-CoV-2- RNA^+^/dsRNA^+^ by FISH-flow. Healthy donor platelet samples were used to establish the threshold of positive detection of infected Vero cells. The percentages of SARS-CoV-2- RNA^+^/dsRNA^+^ Vero cells were converted into PFU using a standard curve (Figure S8D).

Importantly, platelet-free SARS-CoV-2 detected in plasma from non-survivors is non-infectious**.** When the viral content in PPP was evaluated on Vero cells, no PFU could be measured, both in plasma from survivors and non-survivors (Figure S10E), in contrast with bronchoalveolar lavages (BAL) from SARS-CoV-2 patients (Figure 4F, middle graph: virus (green circle)). This adds to current data in literature showing that virus when detected in the plasma is non-infectious (13-15). It is also possible that the RT-qPCR signal detected in the plasma might not represent full viral particles but only free viral RNA components, therefore not infectious or virus in which viral replication cycle could be aborted or blocked later on without the formation of infectious virus.

***In vitro* outgrowth of SARS-CoV-2 from platelets**

Macrophages were obtained and differentiated in vitro using M-CSF, IL-4 and IL-13 as described (1), to generate tissue-like macrophages. Macrophages were seeded in 12-well plate (Corning), at density of 10^6^ cells per well, and cultivated in RPMI medium supplemented with 12.5 ng/ml M-CSF (R&D Systems) and 10% FCS (complete medium) at 37°C, 5% CO_2_. Platelets were added to macrophages cultures by adding 100 μl of PRP into 400 μl macrophage complete medium, and their interaction was carried out overnight at 37°C, 5% CO_2_ (pulse) until non-adherent, non-internalized platelets were washed out by extensive washes using warm complete medium. PRP were pre-treated or not with Abciximab (10 μg/ml, Reopro, Janssen-Cilag B.V., Netherlands) for 15 min at 37°C before being added to macrophage cultures. In some experiments, 20 μl of PRP were added to macrophages seeded in ibidi 8-well removable chamber glass slide (ibidi, GmbH), at density of 5x10^5^ cells per well, 200 μl of complete medium per well, and fixed with 10% NBF after 2-hour pulse and 24-hour chase periods to proceed to RNAscope *in situ* hybridization. After the interaction period (pulse), macrophages were kept in culture in complete medium, which was collected for after 24 hours (chase). Next, 180 μl of collected macrophage supernatants were added to Kidney epithelial cell line from African green monkey (Vero E6) cells cultivated in glass slide with 8-well removable chamber (ibidi, GmbH)(density of 5x10^4^ cells per well, 70% confluent) together with 20 μl of TPCK-treated trypsin (Sigma-Aldrich, final concentration of 1 μg/ml). After 24 hours incubation, the Vero cell cultures were fixed in 4% paraformaldehyde (PFA, Electron Microscopy Sciences, US) and proceeded to indirect immunolabeling of double-strand RNA (dsRNA). For the detection of infected Vero cells were treated with blocking buffer containing 50 mM EDTA (pH=8), 0.45% gelatin from cold-water fish skin (Sigma-Aldrich), 10 mg/ml bovine serum albumin fraction V (ROCHE), 1% horse serum (Vector Laboratories) and 5% human serum (Sigma-Aldrich) (blocking solution) for 2 hours at room temperature. Next, cells were incubated with a mouse IgG2a anti-dsRNA monoclonal antibody (J2, Scicons) at 1:500 v/v concentration in blocking buffer solution, overnight at 4 °C, followed by incubation with secondary anti-mouse IgG-Cy3 (Jackson, 115-166-072) for 2 hours at room temperature. After counterstaining by DAPI, images of immunostained Vero cells were acquired at the Confocal microscope system and images were processed by Imaris software to threshold out non-specific dsRNA signal (using non-infected Vero cells as negative controls). All fields containing specific dsRNA signal were acquired and the number infected cells per well was counted. The total number of Vero cells per well was quantified automatically by Imaris software, building isospots for Vero cell nuclei in entire well images.

In infection experiments in the absence or presence of abciximab, infection was quantified using FISH-flow that combines detection of the J2 signal with viral RNA. 1x10^5^ Vero E6 cells were seeded in 48-well plate and cultivated to reach confluence. 200 μl of the cell culture medium from the macrophages and PRP coculture described above were added into Vero E6 with the presence of TPCK-treated trypsin. After 24 hours incubation, cells were detached with Trypsin for 5 min, fixed in 4% PFA for 30 min and processed as above for FISH-flow quantification. Briefly, fixed cells were incubated with first J2 antibody (1:500 v/v in permeabilization buffer) for 2 hours and the secondary goat anti-mouse IgG2a-FITC (1:8000 v/v in permeabilization buffer, 115-095-206, Jackson) for 1 hour at room temperature. After 2 times wash in hybridization wash buffer, cells were resuspended in the solution which contain the 4 probe sets of SARS-CoV-2 (12.5 nM each) overnight at 37 °C. After two washes in hybridization wash buffer, positive events were acquired by flow cytometry (Guava easyCyte 12HT base system, Millipore).

**Macrophage infection by SARS-CoV-2 detected by Focus Forming Assays (FFA)**

In order to assess infectious particles in macrophage supernatants, monocyte-derived macrophages were infected with SARS-CoV-2 at MOI 1, 0,1 or 0,01 for different periods of time at 37°C without agitation. Macrophage supernatants were harvested and used to directly infect Vero cells cultivated in 12-well plates for Focus Forming Assay (FFA). Briefly, supernatants were added to confluent Vero cell monolayer for 2 hours before removing the inoculum and replacing it with immobilizing media composed of Dulbecco's Modified Eagle Medium (DMEM) supplemented with Low Melting agarose (Sigma-Aldrich A0701) at 0.3% w/v final concentration, from a pre-made 2.4% w/v stock solution. Plates were let sit at room temperature for 15-20 min to allow the agarose to solidify before putting back in the 37°C incubator. After 36 hours incubation, plates were fixed in 4% PFA for 1 hour then permeabilized with solution of PBS supplemented with 0.1%TritonX-100 for 15 min. Vero cells were then immunostained for SARS-CoV-2 using Anti-N Biotinylated antibody (BiossUSA 7E1B, dilution 1:1500 v/v) followed by staining with Streptavidin-HRP (Beckman Coulter PN IM0309, Dilution 1:5000 v/v) and HRP substrate TMB True Blue (Eurobio Scientific, 5510-0030, according manufacturer instructions). Staining steps were carried in PBS supplemented with 0.1% v/v Tween-20 and 2% v/v FCS for at least 45 min at 37°C, with 3-times washing steps between each staining step. Foci were then counted in technical replicates by visual inspection. Infectious Titer presented in the graphs was calculated from this counting as described (16).

**Flow cytometry of platelet and macrophage samples**

Platelets were immunolabelled for vWF by centrifuging 30 μl of PRP at 1100 g, 10 min, and resuspending the platelet pellet in 1% PFA, 30 min for fixation. Platelets were then labelled in suspension by indirect immunofluorescence using 10 μg/ml rabbit polyclonal anti-vWF (abcam, ab6994) followed by 1:200 v/v anti-rabbit secondary antibody coupled to FITC (Jackson, 711-096-152). Next, CD41 immunolabeling was performed using an APC-tagged anti-CD41 antibody (Biolegend, 303710) at 1:50 v/v concentration. Platelets were then analyzed by flow cytometry (Guava easyCyte 12HT base system, Millipore) using a gating strategy consisting in FSC/SSC gating, doublets exclusion and assessment of vWF^+^ platelets in the population of CD41^+^ events. The left-over of flow cytometry analysis was placed in a slide, covered with a coverslip and observed under the confocal system for acquisition of images in order to localize vWF signal associated with platelets. Von Willebrand factor localized at the platelet surface and associated with a higher percentage and intensity (MFI) in non-survivors compared to survivors (Figure S4A-B). In non-survivors, vWF mainly coats the platelet. This is in line with higher vWF plasma concentration and increased vWF surface exposition on platelets from non-survivors as compared to survivors (Table 1).

In addition, platelet activation in fresh platelets samples as compared with freeze-thaw samples was quantified by flow cytometry after platelet fixation and immunolabeling with anti-Human CD42b (BD 555473) and anti-human CD62P (Biolegend 304904) at 1:20 v/v concentration, for 15 minutes at room temperature.

Macrophages were fixed with 4% PFA for 30 min, washed in PBS and incubated for 1 hour, room temperature, with primary antibodies against human ACE2 (10 μg/ml, orb10029 Biorbyt) or TMPRSS2 (1:10 v/v, PA5-14264 ThermoFisher Scientific), followed by incubation for 1 hour, room temperature, with 1:200 v/v anti-rabbit secondary antibody coupled to FITC (Jackson, 711-096-152); both primary and secondary antibodies diluted in permeabilization buffer containing 0.2% v/v Tween-20. Next, macrophages were stained with 1:40 v/v anti-CD68-BV711 in permeabilization buffer for 15 minutes at room temperature before flow cytometry analyses.

**Multiparametric flow cytometry**

***For bronchoalveolar lavage (BAL) cells:*** A 12-channel multiparametric flow cytometry was devised for the detection of platelets, megakaryocytes and macrophages forming conjugates with platelets in BAL, and whether they contain SARS-CoV-2 spike protein. BAL cells were thawed from frozen aliquots, fixed with 4% PFA for 30 min, washed in PBS containing 200 μg/ml of bovine serum albumin (PBS-BSA), and incubated for additional 30 min with 2 μg/ml mouse IgG1 anti-SARS-CoV-2 spike antibody (Genetex, GTX632604) in permeabilization buffer containing 0.2% v/v Tween-20, followed by incubation of 1:1000 v/v secondary anti-mouse IgG1-Cy5 (Southern, 1070-15) diluted in the same permeabilization buffer. After successive washes with PBS-BSA, cells were incubated for 30 minutes, room temperature, with the following antibodies coupled to fluorophores diluted in permeabilization buffer: 1:400 v/v anti-CD15 eFluor450, 1:40 v/v anti-CD41a-BV510, 1:40 v/v CD326 (epCAM)-AlexaFluor488, 1:400 v/v anti-HLADR-Pe-Cy5.5, 1:40 v/v anti-CD68-BV711, 1:20 v/v anti-CD206 PE-Cy7 and 1:200 v/v anti-CD14 APC-AlexaFluor750. Flow cytometry gating strategies for the assessment of frequencies of platelets, megakaryocytes and macrophage-platelet conjugates among BAL cells (infected or not by SARS-CoV-2) are shown in Figure S7C.

***For peripheral blood mononuclear cells (PBMC):*** PBMC were thawed from frozen aliquots, fixed with 4% PFA for 30 min, washed in PBS-BSA and incubated for additional 15 min with the following antibodies coupled to fluorophores diluted in PBS-BSA: 1:40 v/v anti-CD42b BV421, 1:40 v/v anti-CD41a-BV510, 1:20 v/v anti-CD3-APC, 1:20 v/v anti-CD45-FITC, 1:20 v/v anti-CD34-PE-Cy7, 1:20 v/v anti-CD19 APC-H7 and 1:20 v/v anti-CD14-PE. Flow cytometry gating strategy for the assessment of frequencies of megakaryocytes among PBMC is shown in Figure S5A.

**Histology and Immunohistochemistry**

Tissues were immediately transferred to a biological class 2 cabinet and dissected into large pieces after autopsies. Samples were fixed in 10% NBF for at least 24 hours, dehydrated, and paraffin-embedded for further analysis. For each tissue, a minimum of 15-20 sequential sections were cut to perform histology as well as confocal microscopy, as described below. Sections from each block were prepared for hematoxylin and eosin (H&E) and saffron staining, and the stained slides were imaged at 20Å~ using a Hamamatsu slice scanner NanoZoomer 2.0RS. This equipment allows us to scan large tissue areas in the x, y, and z planes. An experienced pathologist reviewed the images and 3D reconstructions. Immunohistochemistry for vWF was performed using rabbit polyclonal anti-human vWF antibody (Dako N1505), 2x diluted, using a Dako Autostainer (Agilent) and following the manufacturer’s instructions.

***In situ* Hybridization of post-mortem tissue**

Bone marrow and lung tissue sections were cut and processed, as described recently (17). Briefly, in addition to deparaffination, we eliminated or reduced autofluorescence by using a light source in the green, and red channel, and by incubating tissues in Sudan Black and sodium borohydride to reduce autofluorescence and for antigen retrieval, as we described (17). The bone marrow and lung tissue sections were treated with for RNAscope Multiplex Fluorescent Reagent Kit v2 Assay protocol (ACD), following manufacturer instructions and adjusting the time for RNAscope Target Retrieval Reagent treatment (322000, ACD) to 15 min and the time for RNAscope Protease Plus Reagent (322380, ACD) treatment to 15 min. Sample were incubated with RNAscope Probe-V-nCoV2019-S-sense (845701, ACD) and hybridization signal was amplified then magnified by TSA Plus cyanine 5 fluorophore, Opal 690 (Akoya Biosciences). Next the samples were incubated in blocking solution for 2 hours at room temperature and then in diluted primary anti-CD41 antibodies (mouse abcam # ab11024, 5μg/ml) overnight at 4 °C. Cells were washed several times with PBS at room temperature and incubated with the Opal 568-coupled secondary antibody for 2 hours at room temperature followed by another wash in PBS. Tissues were examined using an A1 Nikon confocal microscope with spectral detection (Nikon, Japan). Antibody specificity was confirmed by replacing the primary antibody with a non-specific myeloma protein of the same isotype or non-immune serum, as we described and suggested (18).

Analysis of the three-dimensional (3D) reconstruction and deconvolution was performed using NIS Elements (Nikon, Japan), from 12 to 25 optical sections obtained at 0.250- or 0.150-μm intervals. To analyze and quantify the numbers, cellular and nuclear size of lung megakaryocytes specific regions of interest were identified based on CD41 expression. We quantified CD41 positive cells with multi-lobular nuclei (DNA connected), double lobular, and single lobular (micromegakaryocytes).   Also, CD41 negative cells were quantified as a control. To quantify the nuclei (mostly multi-lobular) 3D-deconvoluted optical reconstructions were used. Adjacent sections were stained with hematoxylin to correlate the confocal findings with histopathology of the same tissue sections.

Infected megakaryocytes were quantified based on colocalization of SARS-CoV-2 RNA probe in multilobed CD41 positive cells. No detection of (-) SARS-CoV-2 RNA was detected in uninfected lungs or bone marrow samples. Samples from n=3 individuals with 90 field per samples were analyzed. Kruskall-Wallis H test was performed to determine significance.

To estimate SARS-CoV-2 content of platelets in tissue autopsies, tissues were stained for CD41, SARS-CoV-2 RNA and DAPI as described above. Using fluorescence signal masking prior to Pearson’s signal correlation analysis (19), the signal detected in large CD41+ polylobed MKs was excluded and the resulting image, comprising anucleated CD41+ bodies, was analyzed for colocalization between CD41 and SARS-CoV-2 RNA. Thus, most of the signal remaining after masking out CD41+ megakaryocyte signal corresponded to small CD41+ structure with the size of platelets (1.2 to 2.5 μm). Pearson’s correlation coefficients for colocalization were determined in n=3 different individuals per COVID- and non-COVID condition, with 24-64 fields per tissue analyzed. Kruskall-Wallis H test was performed to determine statistical significance.

To quantify megakaryocyte abundance and diameter size in lung and bone marrow samples stained with hematoxylin, megakaryocytes were identified as cells at least 50 μm in diameter, polylobular nucleus, and nucleus cytoplasm ratio 0.5. Megakaryocytes/megakaryoblasts were counted in different tissue sections as described (20, 21). Control lung autopsy samples were obtained from individuals with cancer (n=3) and tuberculosis (n=3). These control tissues correspond to the border of the tumor or granuloma areas.

**Electron Microscopy**

Fresh platelet samples were immediately fixed in 10 volumes of paraformaldehyde (1.5%) and glutaraldehyde (0.25%) in phosphate buffer, pH7.4 for 30min at room temperatures. Platelets were washed and resulting pellet embedded in Epon and processed for standard electron microscopy as we described (1). Immunogold detection of SARS-CoV-2 spike on ultrathin cryosections was performed as described (22) using 20 or 50 μg/ml anti-spike antibodies (polyclonal rabbit anti-SARS-CoV-2 spike (Genetex GTX135356) or monoclonal mouse IgG1 anti-SARS-CoV-2 spike, Genetex, GTX632604) or a mouse IgG1 isotype control (Becton 349040) at same concentrations. Labeling observed was concentration dependent (Figure S3E). Image acquisition was performed using a JEOL 1011 equipped with a GATAN Orius SC 1000 camera at the Electron Microscopy Platform at the Cochin Institute, and at least 200 fields of about 20-25 platelets were observed per platelet sample. All COVID-19 samples used for ultrastructure were analyzed in parallel for the presence of SARS-CoV-2 by FISH-Flow and after divulgation of the patient outcome were found to come from non-survivors.

**Multiplex cytokine detection assay**

After thawing frozen aliquots of BAL fluid, samples were processed for Multiplex detection of the following cytokines, according to the distributor’s instructions: IL-1α, IL-1β, IL-6, S100A8, CXCL4 and d-dimer (R&D Luminex, R&D) and IFNα, IFNβ, IFNγ, FGF2, PDGFBB, VEGF-A and VEGF-B (Procarta Multiplex, Thermo Scientific). Samples were analyzed in a Bio-Plex 200 system (Bio-Rad) following manufacturer’s instructions.

**Single-cell RNA dataset analysis**

***Integration of single-cell RNA sequencing data:*** Single-cell RNA sequencing data was downloaded from publicly available datasets (23, 24). Cell identification performed by both studies was confirmed by our analysis. The data from Ren X., *et al*. were subset individually to ensure only megakaryocytes characterized by PFA, PPBP, GP9, SPARC, TUBB1 were sampled in the downstream analysis resulting in the selection of 19,568 cells originating from PBMC samples from 30 healthy donors, 140 COVID-19 survivor patients and 13 COVID-19 non-survivor patients for further analysis. Following this step, both datasets were merged and integrated using the Seurat V4 integration pipeline (25). Briefly, the top 2000 variable features were normalized across the two datasets to identify overlapping populations of megakaryocytes. Following, the variable integrated genes were subjected to log_2_ normalization and Principal Component Analysis (PCA). The significant PCA tests were then used to generate a UMAP. Classical and non-classical megakaryopoiesis gene expression was defined by using the function AddModuleScore in Seurat V4 (25). Briefly, genes previously shown to be expressed in classical and non-classical megakaryocyte differentiation (26) were averaged and represented as a score. The scores were then projected on a UMAP of megakaryocytes. The cells were clustered using shared nearest neighbor clustering. We further focused our analysis on singlets, best characterized by canonical megakaryocyte genes (Figure 2B and Figure S5B-D). Technical doublets were discarded based on expression of genes canonical for other cells and also based on increased mRNA content (Figure S5C-D). Differentially expressed genes (DEGs) among clusters were identified using a Wilcoxon Rank Sum test. DEGs were selected based on an FDR value less than 0.05 and a log_2_FC greater than 0.25. Volcano plots were generated using SPSS software.

***Trajectory Analysis:*** After identification of megakaryocyte clusters using Seurat V4, we implemented the package Monocle 3 (27) to predict cell transitions over pseudotime. Significant gene transitions were identified by a spatial correlation analysis. Genes for heatmapping were selected based on a *p* value less than 0.05 and a Morans I score greater than 0.25.

***Gene Ontology Analysis:*** Gene ontologies were identified from uploading differentially expressed gene lists from each megakaryocyte cluster to the online tool Metascape (28). Only gene ontologies with an FDR less than 0.05 are shown.

**Statistical analyses**

Summary statistics, median with interquartile range [IQR] and percentages, are reported. Comparison across hospital outcome groups used the exact Fisher test and the Wilcoxon rank sum test. Univariate and multivariate logistic regression models were used to assess predictors of hospital mortality, with odds ratio (OR) and 95% confidence intervals [95%CI] used as the measure of association with the outcome. All tests were two-sided with p-values of 0.05 or less denoting statistical significance.

The results in graphs are represented as boxplots or scatterplots with individual values represented as dots, or as mean with standard errors. Statistical tests were performed considering normal (parametric tests, Student’s t-test or ANOVA with pairwise comparisons) or non-normal distributions (non-parametric tests, Mann-Whitney or Kruskal–Wallis). Paired-samples T-test was performed to compare same samples in different time points. Bivariate correlations were assessed by two-tailed Pearson correlation coefficients. Significant differences were indicated by asterisks, considering *p* values below 0.05.

**Supplementary References**

1. Real F, Capron C, Sennepin A, Arrigucci R, Zhu A, Sannier G, et al. Platelets from HIV-infected individuals on antiretroviral drug therapy with poor CD4(+) T cell recovery can harbor replication-competent HIV despite viral suppression. Sci Transl Med. 2020;12(535).

2. Cramer E, Lu H, Caen J, Soria C, Berndt M, Tenza D. Differential redistribution of platelet glycoproteins Ib and IIb-IIIa after plasmin stimulation [published erratum appears in Blood 1991 Jul 15;78(2):545]. Blood. 1991;77(4):694-9.

3. Delclaux C, Roupie E, Blot F, Brochard L, Lemaire F, Brun-Buisson C. Lower respiratory tract colonization and infection during severe acute respiratory distress syndrome: incidence and diagnosis. Am J Respir Crit Care Med. 1997;156(4 Pt 1):1092-8.

4. Hierholzer JC, Killington RA. 2 - Virus isolation and quantitation. In: Mahy BWJ, Kangro HO, editors. Virology Methods Manual. London: Academic Press; 1996. p. 25-46.

5. Dias SSG, Soares VC, Ferreira AC, Sacramento CQ, Fintelman-Rodrigues N, Temerozo JR, et al. Lipid droplets fuel SARS-CoV-2 replication and production of inflammatory mediators. PLoS Pathog. 2020;16(12):e1009127.

6. Schonborn J, Oberstrass J, Breyel E, Tittgen J, Schumacher J, Lukacs N. Monoclonal antibodies to double-stranded RNA as probes of RNA structure in crude nucleic acid extracts. Nucleic Acids Res. 1991;19(11):2993-3000.

7. Moustafa A, Aziz RK. Traces of SARS-CoV-2 RNA in the Blood of COVID-19 Patients. medRxiv. 2020:2020.05.10.20097055.

8. Deleage C, Wietgrefe SW, Del Prete G, Morcock DR, Hao XP, Piatak M, Jr., et al. Defining HIV and SIV Reservoirs in Lymphoid Tissues. Pathog Immun. 2016;1(1):68-106.

9. Real F, Sennepin A, Ganor Y, Schmitt A, Bomsel M. Live Imaging of HIV-1 Transfer across T Cell Virological Synapse to Epithelial Cells that Promotes Stromal Macrophage Infection. Cell Rep. 2018;23(6):1794-805.

10. Simon AY, Sutherland MR, Pryzdial EL. Dengue virus binding and replication by platelets. Blood. 2015;126(3):378-85.

11. Piersma SR, Broxterman HJ, Kapci M, de Haas RR, Hoekman K, Verheul HM, et al. Proteomics of the TRAP-induced platelet releasate. J Proteomics. 2009;72(1):91-109.

12. Maguire PB, Parsons ME, Szklanna PB, Zdanyte M, Munzer P, Chatterjee M, et al. Comparative Platelet Releasate Proteomic Profiling of Acute Coronary Syndrome versus Stable Coronary Artery Disease. Front Cardiovasc Med. 2020;7:101.

13. Kwon SY, Kim EJ, Jung YS, Jang JS, Cho NS. Post-donation COVID-19 identification in blood donors. Vox Sang. 2020;115(8):601-2.

14. Cappy P, Candotti D, Sauvage V, Lucas Q, Boizeau L, Gomez J, et al. No evidence of SARS-CoV-2 transfusion transmission despite RNA detection in blood donors showing symptoms after donation. Blood. 2020;136(16):1888-91.

15. Andersson M, Arancibia-Carcamo C, Auckland K, Baillie J, Barnes E, Beneke T, et al. SARS-CoV-2 RNA detected in blood products from patients with COVID-19 is not associated with infectious virus [version 2; peer review: 2 approved]. Wellcome Open Research. 2020;5(181).

16. Amarilla AA, Modhiran N, Setoh YX, Peng NYG, Sng JDJ, Liang B, et al. An Optimized High-Throughput Immuno-Plaque Assay for SARS-CoV-2. Front Microbiol. 2021;12:625136.

17. Prevedel L, Ruel N, Castellano P, Smith C, Malik S, Villeux C, et al. Identification, Localization, and Quantification of HIV Reservoirs Using Microscopy. Curr Protoc Cell Biol. 2019;82(1):e64.

18. Rella CE, Ruel N, Eugenin EA. Development of imaging techniques to study the pathogenesis of biosafety level 2/3 infectious agents. Pathogens and Disease. 2014;72(3):167-73.

19. Luu R, Valdebenito S, Scemes E, Cibelli A, Spray DC, Rovegno M, et al. Pannexin-1 channel opening is critical for COVID-19 pathogenesis. iScience. 2021;24(12):103478.

20. Hu B, Tang Y, Chang EI, Fan Y, Lai M, Xu Y. Unsupervised Learning for Cell-Level Visual Representation in Histopathology Images With Generative Adversarial Networks. IEEE Journal of Biomedical and Health Informatics. 2019;23(3):1316-28.

21. Mason RJ. Pathogenesis of COVID-19 from a cell biology perspective. European Respiratory Journal. 2020;55(4):2000607.

22. Youssefian T, Drouin A, Masse JM, Guichard J, Cramer EM. Host defense role of platelets: engulfment of HIV and Staphylococcus aureus occurs in a specific subcellular compartment and is enhanced by platelet activation. Blood. 2002;99(11):4021-9.

23. Bernardes JP, Mishra N, Tran F, Bahmer T, Best L, Blase JI, et al. Longitudinal Multi-omics Analyses Identify Responses of Megakaryocytes, Erythroid Cells, and Plasmablasts as Hallmarks of Severe COVID-19. Immunity. 2020;53(6):1296-314 e9.

24. Ren X, Wen W, Fan X, Hou W, Su B, Cai P, et al. COVID-19 immune features revealed by a large-scale single-cell transcriptome atlas. Cell. 2021;184(7):1895-913 e19.

25. Hao Y, Hao S, Andersen-Nissen E, Mauck WM, 3rd, Zheng S, Butler A, et al. Integrated analysis of multimodal single-cell data. Cell. 2021.

26. Choudry FA, Bagger FO, Macaulay IC, Farrow S, Burden F, Kempster C, et al. Transcriptional characterization of human megakaryocyte polyploidization and lineage commitment. J Thromb Haemost. 2021;19(5):1236-49.

27. Cao J, Spielmann M, Qiu X, Huang X, Ibrahim DM, Hill AJ, et al. The single-cell transcriptional landscape of mammalian organogenesis. Nature. 2019;566(7745):496-502.

28. Zhou Y, Zhou B, Pache L, Chang M, Khodabakhshi AH, Tanaseichuk O, et al. Metascape provides a biologist-oriented resource for the analysis of systems-level datasets. Nat Commun. 2019;10(1):1523.

**Supplementary Figures**


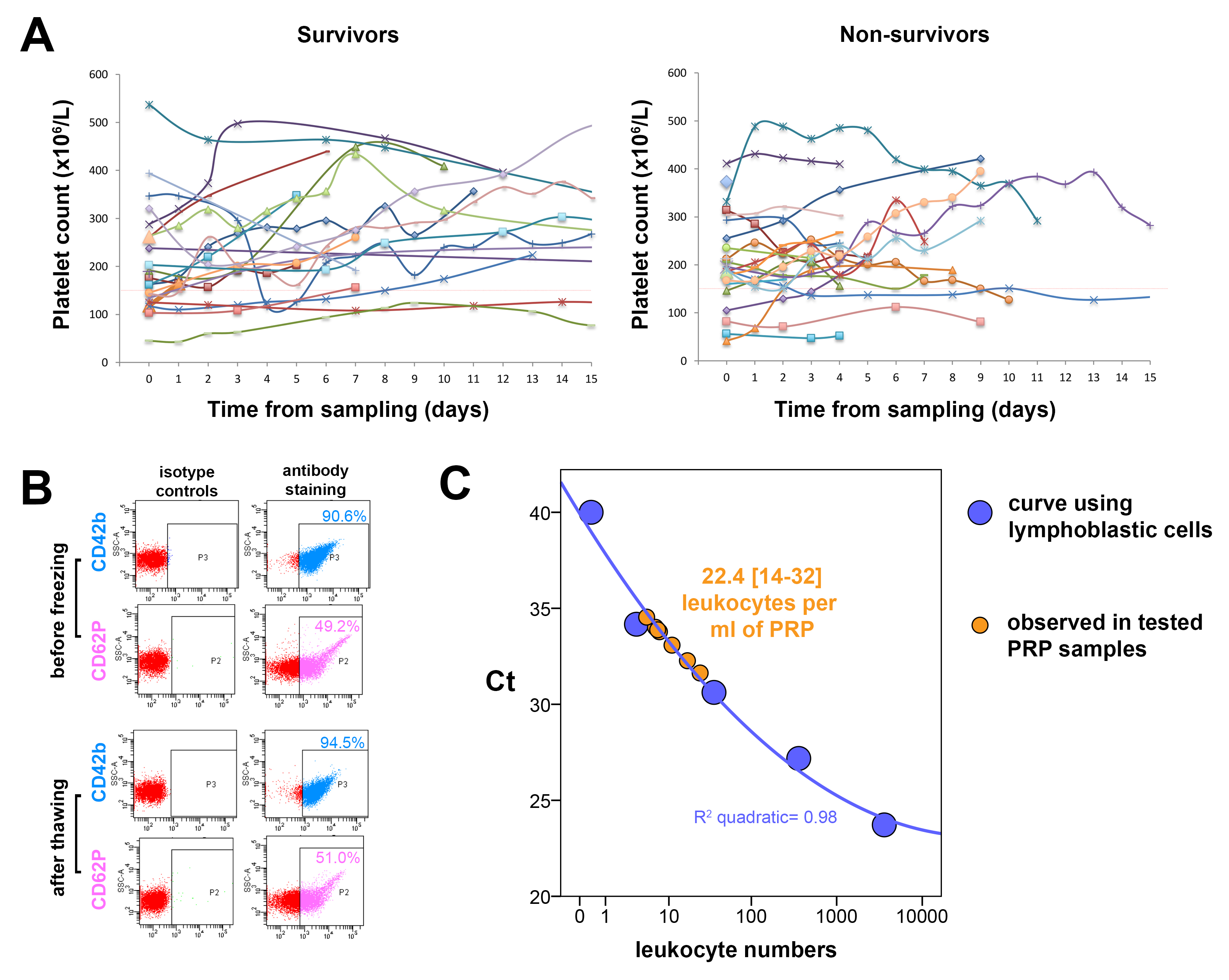


**Figure S1. Supplementary information of platelet samples.**

(A) Longitudinal platelet count from the date of sampling for SARS-CoV-2 in platelets during 15 days for survivors (n= 23 left) or until discharge for non-survivors (n=25, right). Dotted red line indicates the lower limit of normal platelet count.

(B-C) Quality of platelet preparation after freeze-and-thaw procedures:

(B) Percentage of CD42b^+^ (viable, blue dots) and CD62P^+^ (activated, pink dots) platelets in PRP samples before freezing and after thawing from -80°C cryopreservation.

(C) RT-PCR Ct curves for CD45 (PTPRC gene) obtained from different lymphoblastic CEM cell line numbers (blue standard curve), and from PRP samples used to detect SARS-CoV-2 in platelets (orange dots plotted over standard curve). The mean number with 95% confidence interval of leukocytes found per ml of PRP is shown.

**
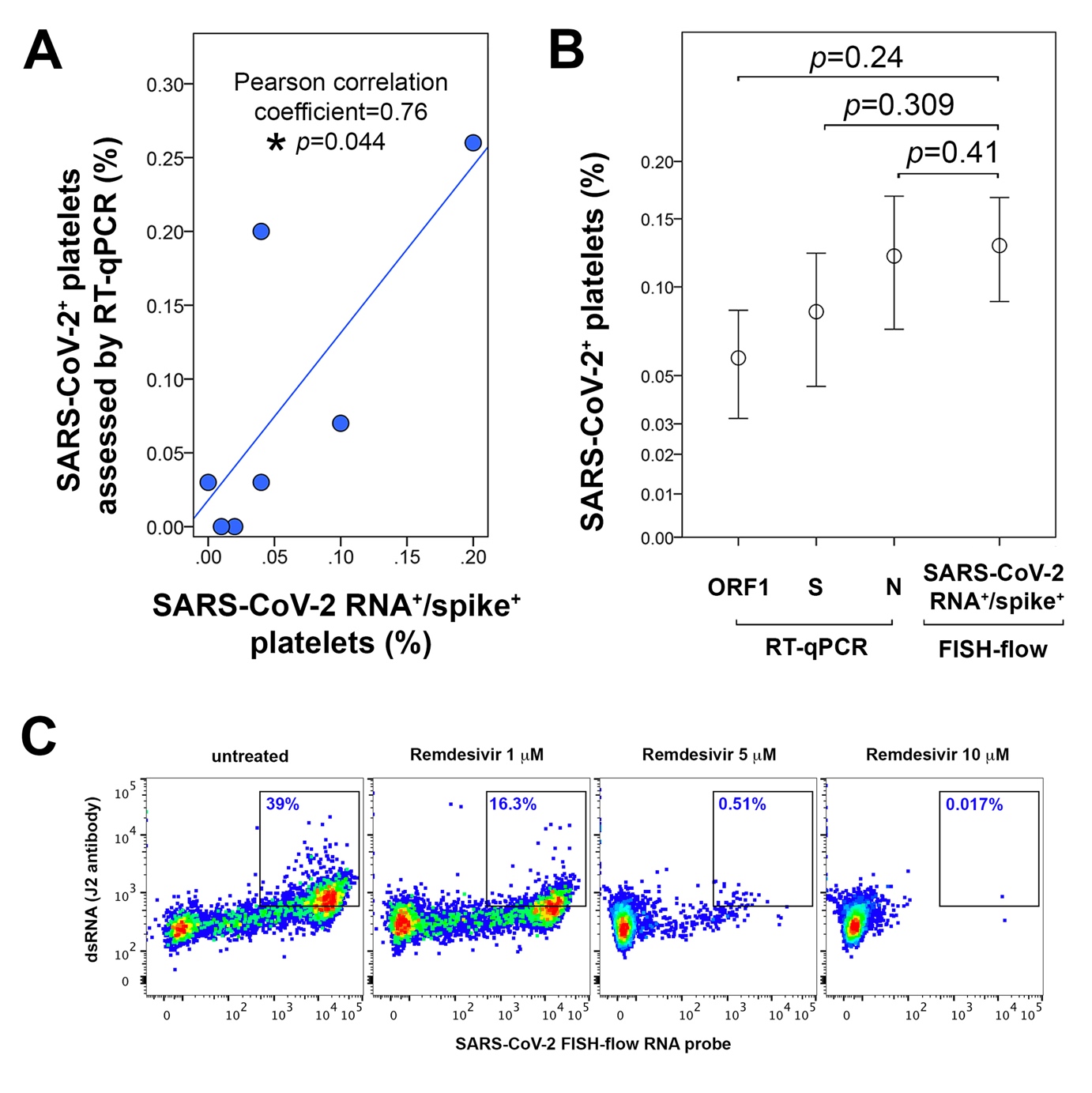
**

**Figure S2. FISH-flow experimental validation.**

(A) Bivariate correlation between Percentages of SARS-CoV-2^+^ platelets among total platelets quantified by RT-qPCR (S gene) and by SARS-CoV-2 RNA/ spike protein FISH-flow. R^2^ coefficient of determination and two-tail Pearson correlation coefficients with its statistical significance.

(B) Percentages of SARS-CoV-2^+^ platelets among total platelets quantified by RT-qPCR (ORF1, S and N gene targets) and by SARS-CoV-2 RNA/ spike protein FISH-flow. Mann-Whitney test comparing pairwise FISH-flow percentages and the percentages quantified by RT-qPCR for each gene target (data represented as mean and standard error).

(C) Combined detection of FISH-flow RNA probes and dsRNA immunolabeling by Flow-cytometry at the single cell level, in Vero cells infected with 5000 PFU for 24 hours, continuously treated or not with the SARS-CoV-2 replication inhibitor Remdesivir (1-10 μM). Gate shows the percentage of SARS-CoV-2 RNA^+^/dsRNA^+^ Vero cells among the entire Vero cell population.

**
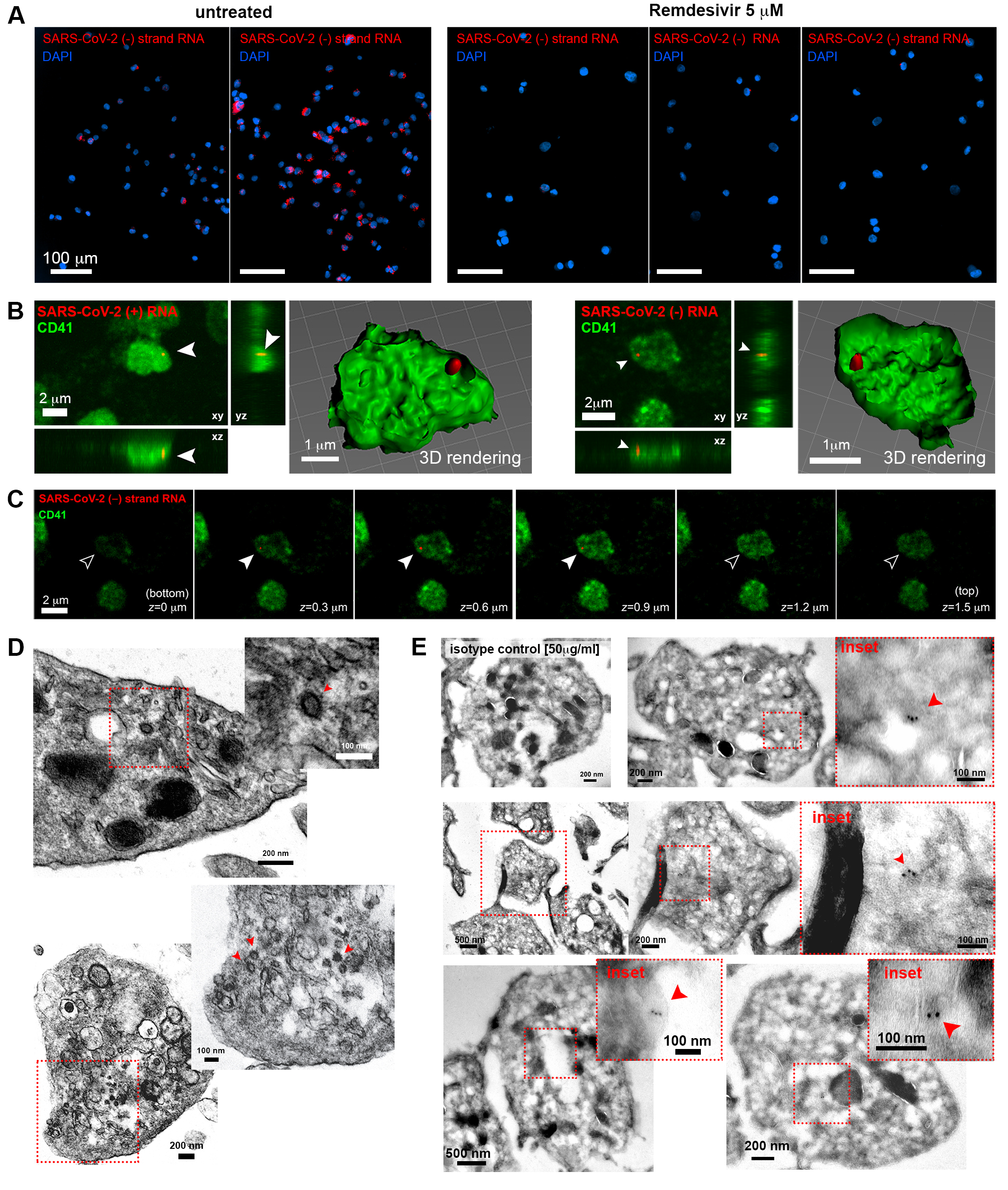
**

**Figure S3. Additional confocal microscopy and electron microscopy images of platelets containing SARS-CoV-2 localized exclusively inside platelets and not at their surface.**

(A) SARS-CoV-2 (-) strand RNA FISH using the RNAscope technique applied to Vero cells infected with 5000 PFU for 24 hours, treated or not with 5 μM Remdesivir. Bar= 100μm.

(B) Confocal microscopy images after CD41 immunolabeling in green and *in situ* hybridization for SARS-CoV-2 (+) RNA (left) and (-) RNA (right) in red in platelet samples from COVID-19 non-survivors. Images show three-dimensional projections (middle - xy, xz and yz, bar=2μm) and three-dimensional rendering after reconstruction (right). Arrowheads indicate SARS-CoV-2 RNA.

(C) Confocal microscopy serial z-stack images taken 0.3 μm apart of the virus-containing platelets from a COVID-19 non-survivor, from z-stack coordinate 0 (bottom) to 1.5 μm (top of the platelet). Platelets are identified by CD41 immunolabeling (green). The SARS-CoV-2 RNA (red) signal is restricted to central z-sections (with z=0.3, 0.6 and 0.9μm) as pointed by white arrowheads. Empty arrowhead in surrounding z-sections (with z=0, 1.2 and 1.5μm) indicate the site where SARS-CoV-2 was detected in central z-sections, although no red signal is detected, indicating that SARS-CoV-2 RNA was localized only inside platelets and not at its surface. Bar= 2μm.

(D) Additional electron microscopy images of platelets containing spherical crowned SARS-CoV-2 particles of 50-80nm in diameter (arrowheads) in fresh (A) or frozen (B) platelet samples of different non-survivor patients (bar= 200nm). Dotted line indicates area magnified as shown in the inset (bar= 100nm).

(E) Cryosection of platelets from non-survivors, immunolabelled for spike protein using monoclonal (mAb) or polyclonal (pAb) antibodies. First image shows a platelet cryosection treated with an irrelevant mAb used as negative control for the immunogold labeling using anti-spike protein immunogold labeling. The following images show two examples of immunogold labeling of spike protein within non-survivor platelets. Dotted squares point magnified regions where spike proteins were immunolocalized (red arrowhead). No spike immunolabeling was observed on platelet surface. In both specific and the control labeling, mitochondria lack immunogold labeling, a usual source of immunogold background. Bar=100, 200 or 500 nm.

**
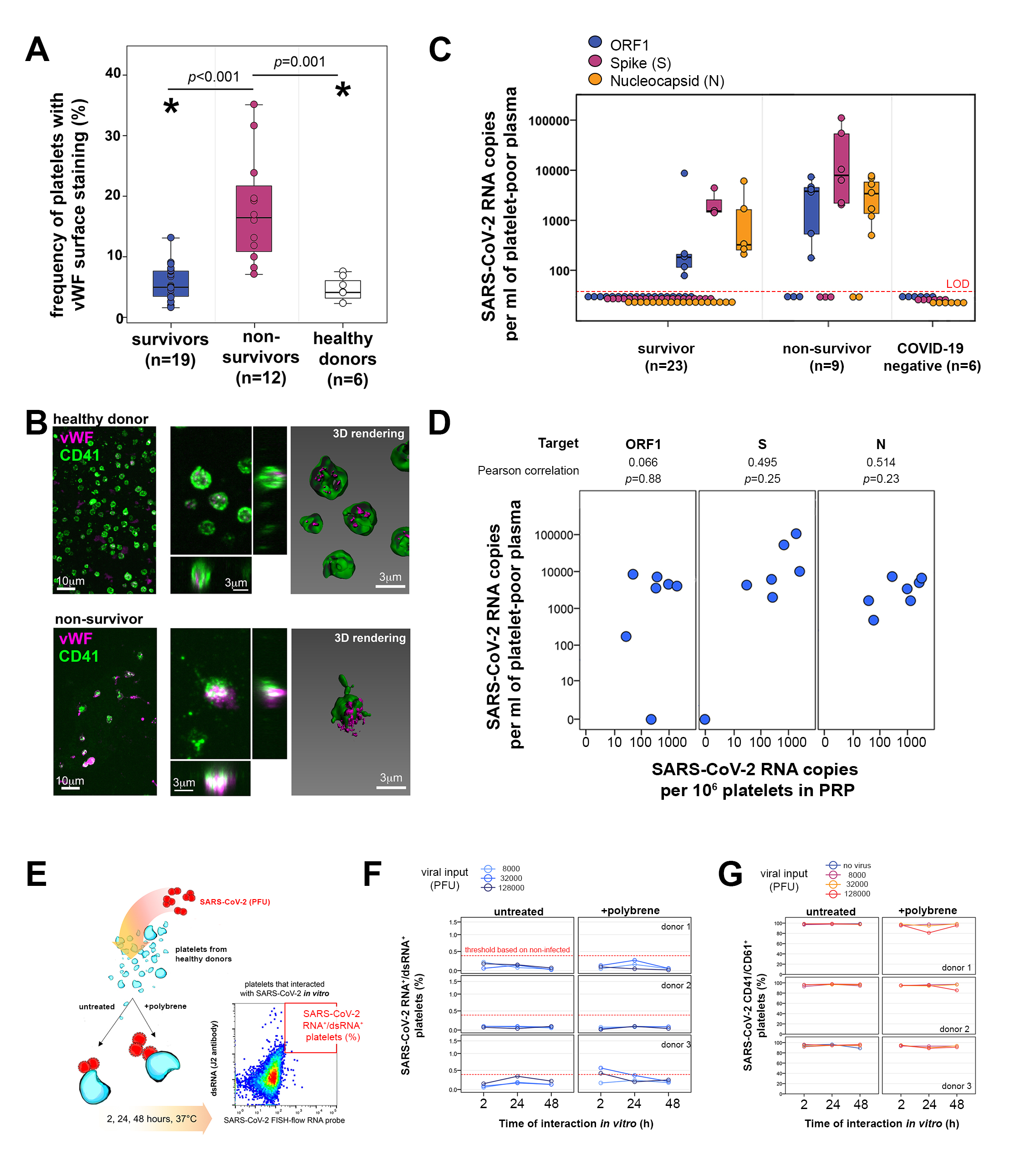
Figure S4. Virus found in the plasma is non-infectious and does not quantitatively correlate with virus found within platelets; these are unable to endocytose SARS-CoV-2.**

(A) Frequency of platelets expressing vWF on their surface among platelets from COVID-19 survivors, COVID-19 non-survivors and healthy donor samples detected by flow cytometry. Asterisk indicates statistical significance in the comparison between survivors and non-survivors and between non-survivors and healthy donors (Kruskal-Wallis between the three groups).

(B) Confocal microscopy images after CD41 (green) and vWF (magenta) immunolabeling using platelet samples from a healthy donor (left) and COVID-19 non-survivor (right). Images show low magnification images (bar=10μm, three-dimensional projections (xy, xz and yz, bar=3μm) and three-dimensional rendering (bar=3μm).

(C) RT-qPCR for SARS-CoV-2 genes ORF1 (blue), S (magenta) and N (orange) in platelet-poor plasma (PPP) samples from severe COVID-19 survivors (n=23), non-survivors (n=9), and COVID-19 negative healthy donor controls (n=6). Results show gene copies per ml of PPP. LOD= limit of detection.

(D) Bivariate correlation between SARS-CoV-2 copies per ml of PPP and SARS-CoV-2 copies per million platelets in platelet-rich plasma (PRP) for each gene target, namely ORF1, S and N. Two-tailed Pearson correlation coefficients with statistically non-significant *p* values are shown.

(E) Scheme of the *in vitro* experiment. SARS-CoV-2 viruses (red) were incubated with platelets for 2, 24 and 48 hours at 37°C in the absence (untreated) or in the presence of polybrene (+polybrene). After extensive washes to eliminate input viruses, platelets were double-labeled for dsRNA and SARS-CoV-2 (+) strand RNA probes and analyzed by flow cytometry (dsRNA/(+)SARS-CoV-2 RNA FISH-Flow) (n=3 independent donors).

(F) FISH-flow percentage of SARS-CoV-2 RNA^+^/dsRNA^+^ platelets among healthy donor platelets (n=3 independent donors) treated or not with polybrene, interacting with different viral inputs for 2, 28 and 48 hours at 37°C.

(G) Percentage of CD41/CD61^+^ platelets in platelet samples tested in the same experiment.

**
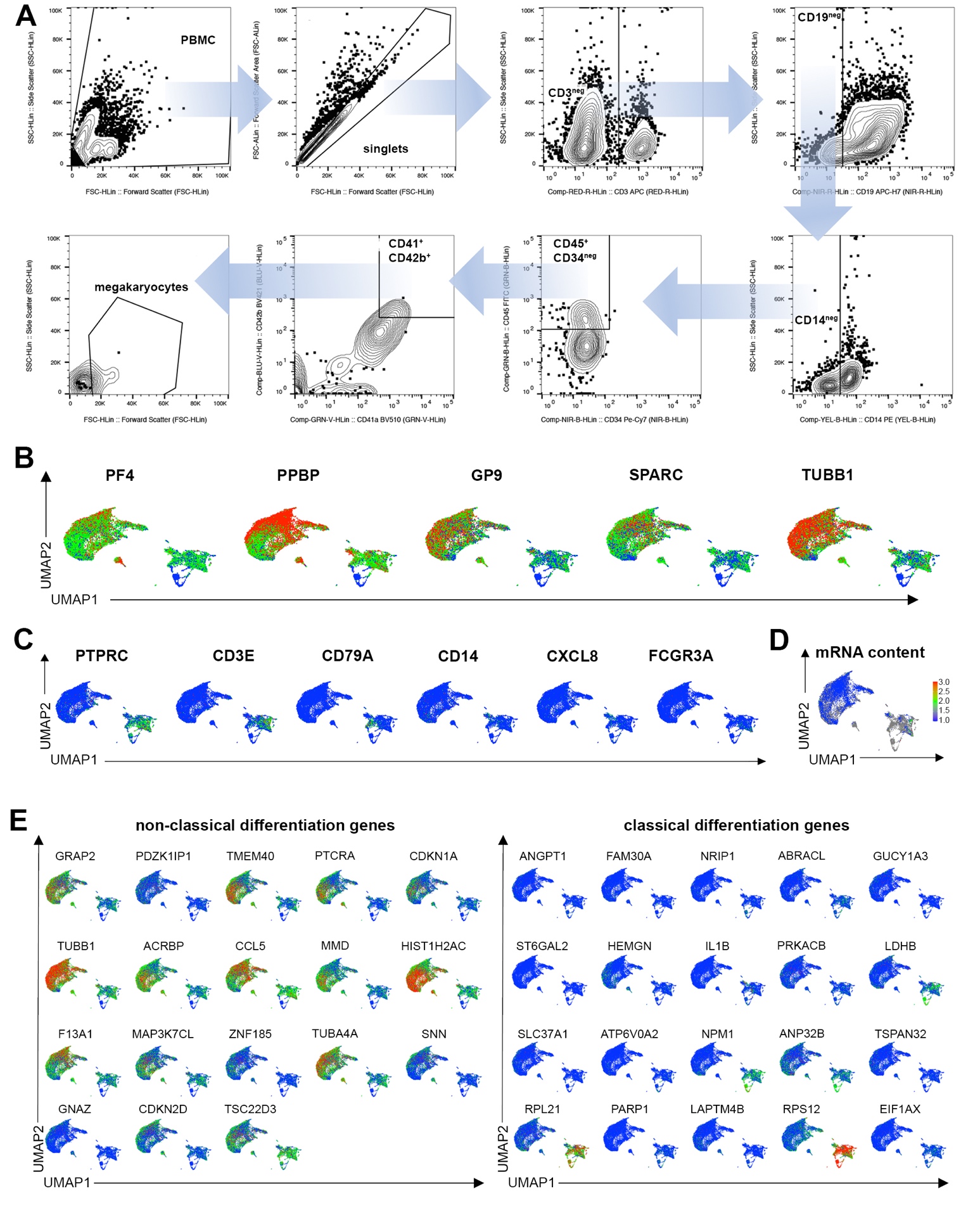
**

**Figure S5. FACS gating strategies and scRNA-seq analytical approach to assess PBMC-derived megakaryocytes.**

(A) PBMC are gated according to FSC and SSC (cells gate), followed by singlets gating and successive CD3^neg^, CD19^neg^, CD14^neg^ gatings. Next, CD3^neg^CD19^neg^CD14^neg^ cells were gated for CD45^+^CD34^neg^ events, followed by CD41^+^CD42b^+^ events. Finally, CD3^neg^CD19^neg^CD14^neg^ CD34^neg^ CD45^+^ CD41^+^CD42b^+^ cells were scored based on size, gating FSC^high^ as megakaryocytes.

(B) Set of genes characterizing with megakaryocytes projected on UMAP built from total cells analyzed.

(C) UMAP displaying leukocyte gene expression: PTPRC (CD45) for leukocytes, CD3 for lymphocytes, CD79a for B cells, CD14 for myeloid cells, CXCL8 (IL-8) for neutrophils, macrophages, epithelial and endothelial cells, and FCGR3A (Fcg-RIIIa) for natural killer cells, macrophages, and subpopulation of T-cells.

(D) UMAP displaying mRNA content in arbitrary units scale (1.0 to 3.0)

(E) UMAP displaying gene expression associated with non-classical and classical megakaryocyte differentiation.

**
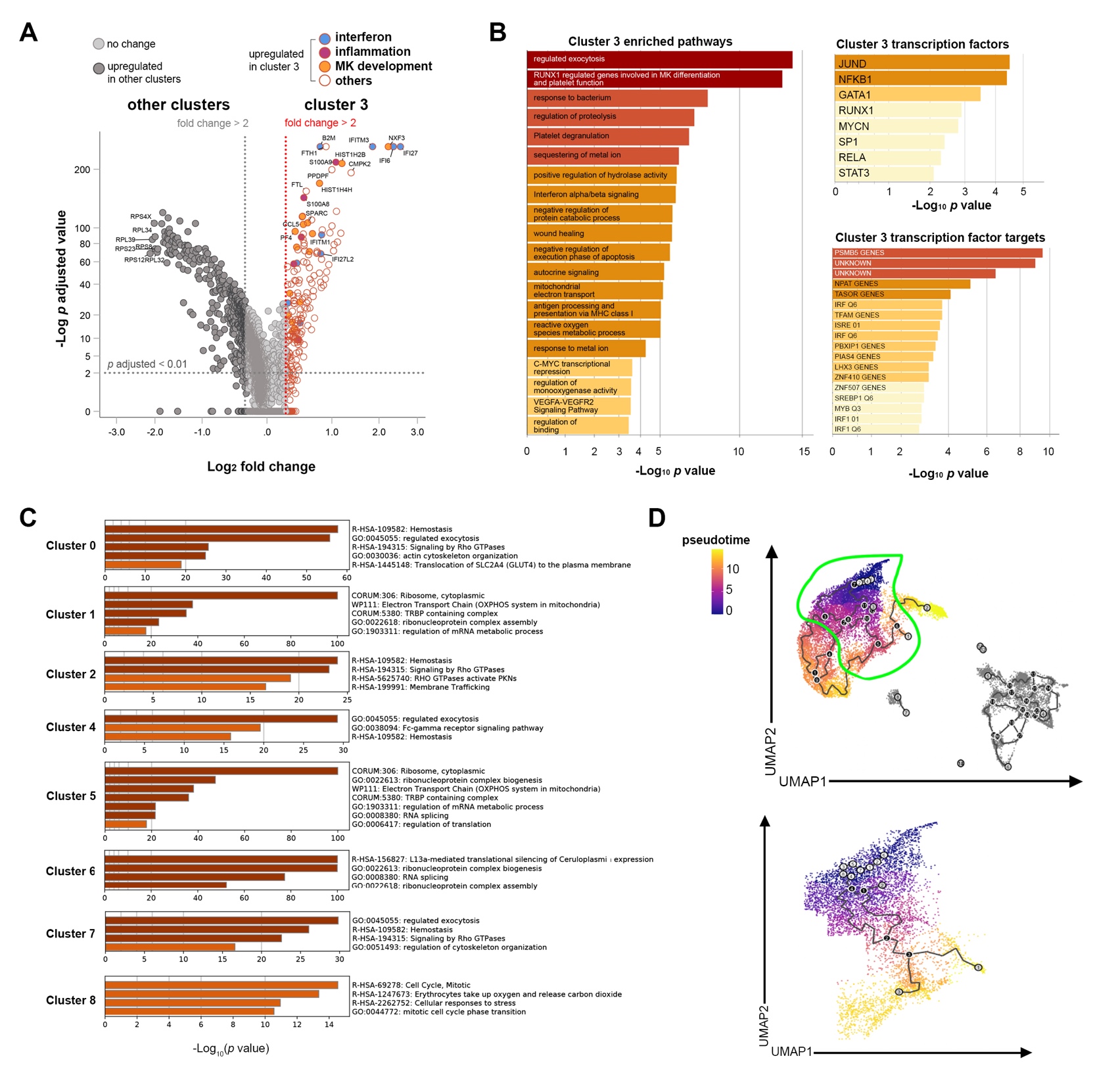
**

**Figure S6**: **Gene expression of** **megakaryocytes employed in clustering analysis.**

**(A)** Volcano plot showing the differentially expressed genes associated with cluster 3 as compared to other clusters. Genes on the left (negative fold change) are increased in all clusters but cluster 3; genes on the right are increased in cluster 3. Some genes upregulated in cluster 3 and related to interferon signalling (blue genes), inflammation (magenta genes), and megakaryocyte development (orange genes) are indicated considering a cutoff of 0.25 log_2_ fold change increase and an adjusted *p* value <0.01.

**(B)** Pathways significantly associated with megakaryocytes in cluster 3, regarding functional pathways, transcription factors and transcription factor targets.

**(C)** Gene ontology pathways enriched in megakaryocytes clusters identified by scRNA-seq sequencing.

Bar graphs showing GO entries and their statistical significance in the enrichment analysis (-Log_10_ *p* value) for DEGs presented by clusters 0, 1, 2, 4, 5, 6,7 and 8 as identified in the clustering analysis of megakaryocytes after scRNA-seq database integration.

**(D)** UMAP of unsupervised trajectory analysis of megakaryocytes initiated at cluster 0 among all UMAP (Top panel); zoom on megakaryocytes area delimited by the green gate allowing to visualize trajectory transitioning from cluster 0 to cluster 3 (Bottom panel).

**
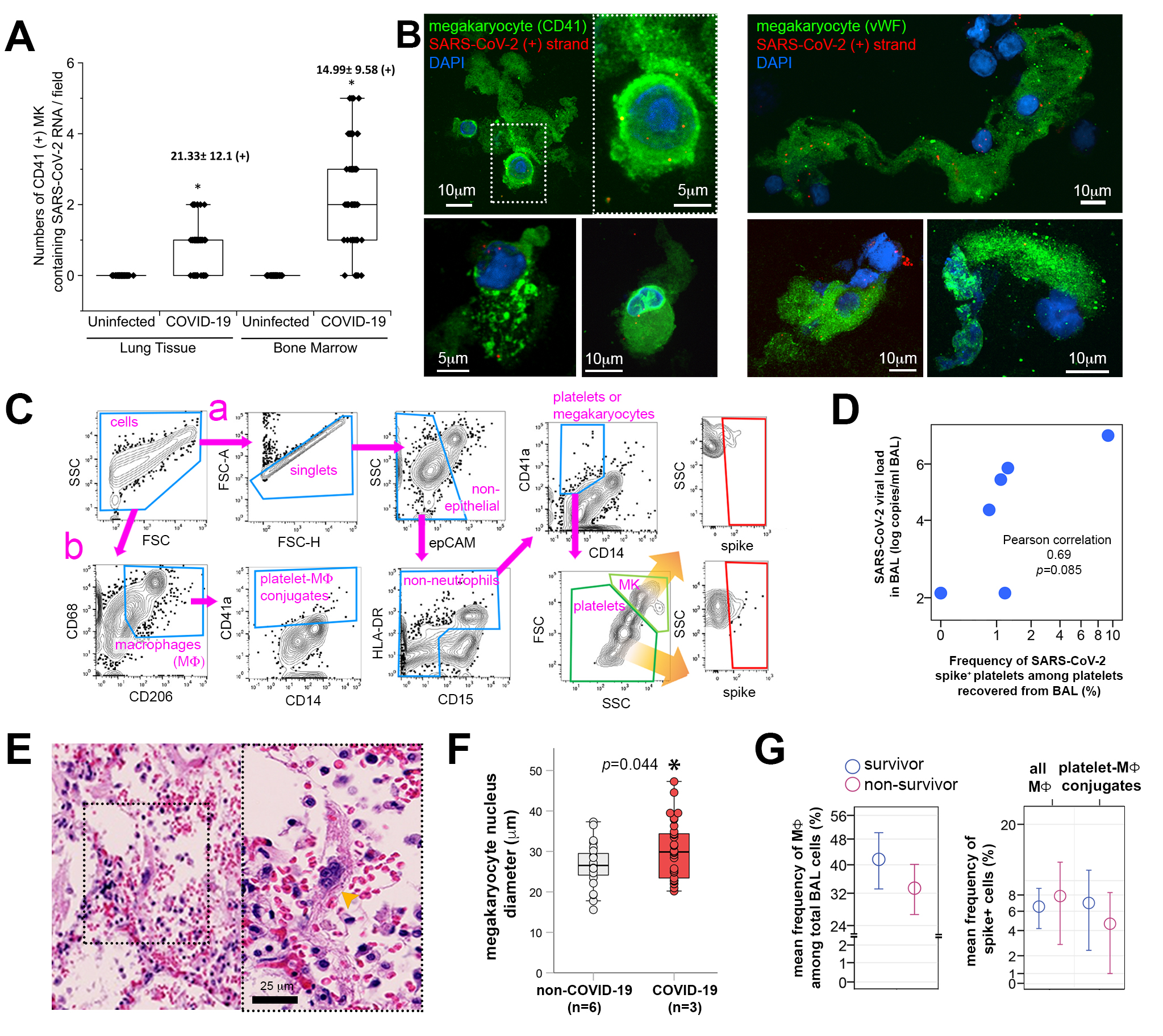
**

**Figure S7. Infected and abnormal lung megakaryocytes accessed in BAL by FACS and confocal microscopy, and in tissues by histology**

(A) Quantification of the numbers of MK (polylobed CD41^+^ cells) in lung and bone marrow tissue sections. Overall, a total of 90 fields per patient were analyzed to reach 24 positive fields with MK positive for (-) SARS-CoV-2 RNA. In the lungs, 21.33± 12.1 % of the MK were positive for (-) SARS-CoV-2 RNA and in the bone marrow, 14.99± 9.58 % of the cells were positive to SARS-CoV-2 RNA. No detection of (-) SARS-CoV-2 RNA was detected in uninfected lungs or bone marrow samples. The Kruskall-Wallis H test, asterisk indicates p≤0.005, n=3 different individuals per condition with 90 fields analyzed).

(B) Confocal images after SARS-CoV-2 RNA *in situ* hybridization (red) for positive (+) strand RNA and immunolabeling of either CD41 (left) or vWF (right), both in green, in BAL samples from two different COVID-19 non-survivors. Arrowheads indicate SARS-CoV-2 RNA inside megakaryocytes. Bar=5 and 10μm.

(C) Flow cytometry gating strategies employed for bronchoalveolar lavage cell suspensions. The first strategy (a) characterizes infected megakaryocytes and virus-containing platelets: from the cell suspension analyzed by forward (FSC) and side scatter (SSC) parameters, singlets are selected by FSC-Area/FSC-Height parameters, followed by exclusion of epCAM^+^ epithelial cells and then neutrophils (HLA-DR^neg^/CD15^+^ cells). The remaining events were analyzed by expression of CD41a and CD14, and platelets and megakaryocytes were gated as (CD14^neg^, CD41a^+^). Next, platelets and megakaryocytes were discriminated by size and complexity using FSC and SSC parameters. Both populations of platelets and megakaryocytes were analyzed according to the presence of SARS-CoV-2 spike protein, with detection gating established using COVID-19 negative BAL controls. The second strategy (b) characterizes platelet-macrophage conjugates: from the cell suspension analyzed by forward (FSC) and side scatter (SSC) parameters, macrophages were assessed by double expression of CD68 and CD206. Next, events were analyzed according to CD41a and CD14 expression, and platelet-macrophage conjugates were assessed in a gate including CD41a^+^ and CD14^+^ or CD41^neg^ events).

(D) Bivariate correlation between SARS-CoV-2 copies per ml of BAL (log_10_ copies/ml BAL) and percentage of spike^+^ platelets among platelets recovered in BAL. Two-tailed Pearson correlation coefficients with statistically non-significant *p* values are shown.

(E) Hematoxylin/eosin histology of lung tissue showing a megakaryocyte indicated by dotted square region (left column), shown in high magnification (right column) inside alveolar space (bar=25μm). Orange arrowheads indicate megakaryocytes.

(F) Measurement of nucleus diameter size of lung megakaryocytes from non-COVID-19 (n=6) and COVID-19 non-survivors (n=3) lung autopsy tissues. Asterisk indicates statistical significance (Mann-Whitney test).

(G) Left: frequency of macrophages, megakaryocytes (MK) and platelets among total BAL cells from survivors (blue) and non-survivors (magenta). No statistically significant differences were found between the two groups (Mann-Whitney test, data represented as mean and standard error). Right: frequency of spike^+^ cells comparing cells from survivors (blue) and non-survivors (magenta). First graph: Frequency of spike^+^ macrophages among all macrophages (all MΦ) and of spike^+^ macrophages among those macrophages forming conjugates with platelets (MΦ+platelets). Second graph: Frequency of spike^+^ megakaryocytes (MK) and platelets among all MK and platelets retrieved in BAL respectively. No statistically significant differences were found between survivor and non-survivor group except for the higher frequency of spike^+^ MK and platelets in non-survivor group (Mann-Whitney test, data represented as mean and standard error).

**
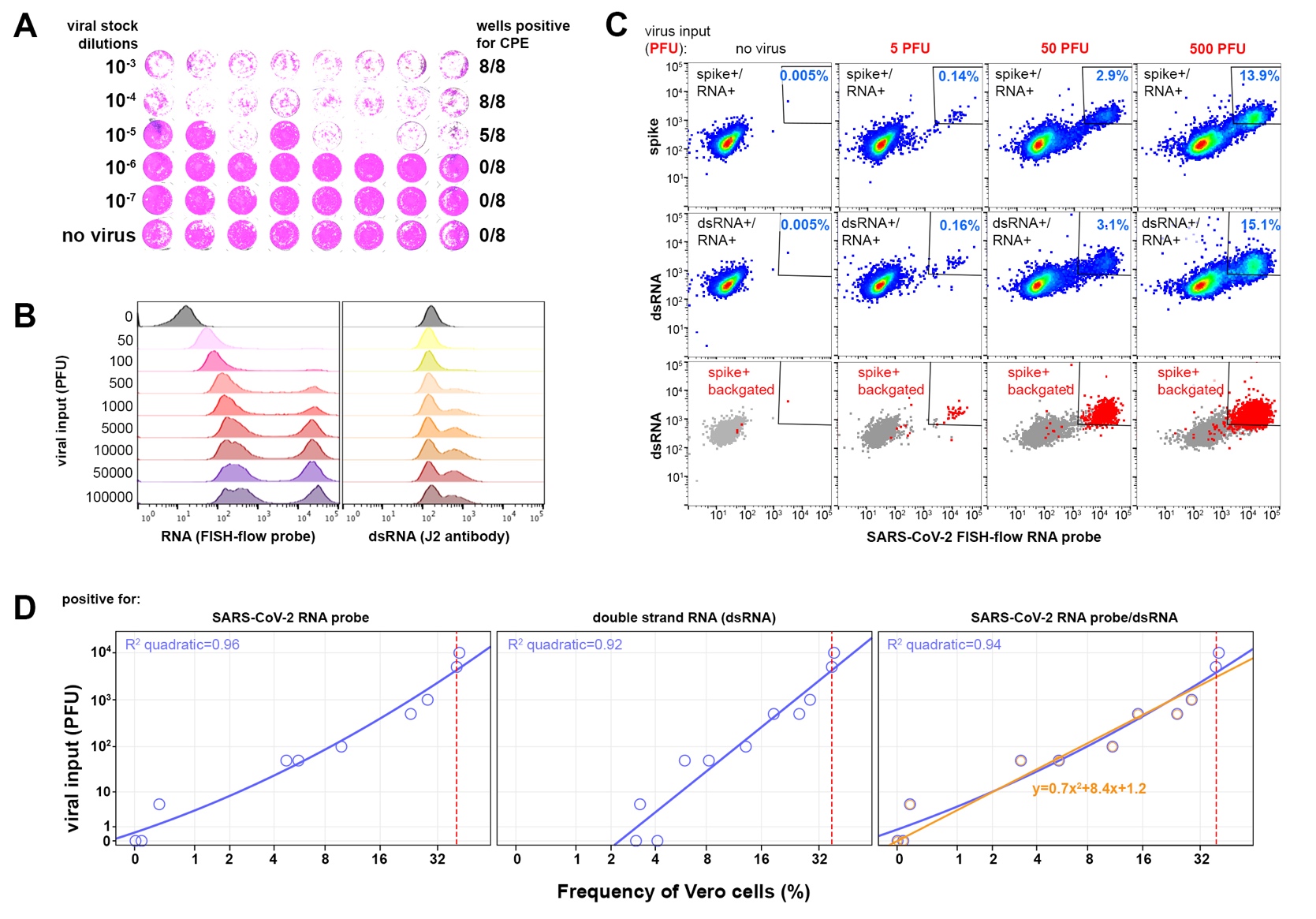
**

**Figure S8. FISH-flow validation using Vero cell infection with titrated primary SARS-CoV-2.**

(A) TCID_50_ titration example of Vero cell culture plate wells stained by Giemsa after infection with different dilutions of a viral stock obtained from severe COVID-19 bronchoalveolar lavage. Wells were scored for the appearance of cytopathic effects (CPE) in 8 replicates, in order to calculate TCID_50_ and conversion into PFU. (B) FACS histograms of FISH-flow (+) SARS-CoV-2 RNA probe and dsRNA immunolabeling signals obtained from Vero cells infected with different PFU of the TCID_50_ titrated viral isolate.

(C) Triple detection of FISH-flow RNA probe, dsRNA immunolabeling and spike immunolabeling by FACS. First row: Combined detection of FISH-flow RNA probe and spike immunolabeling; second row: combined detection of FISH-flow RNA probe and dsRNA immunolabeling; third row: combined detection of FISH-flow RNA probe and dsRNA immunolabeling in which spike^+^ events (in red) were backgated.

(D) Standard curves obtained by correlating the percentage of SARS-CoV-2 RNA^+^ and/or dsRNA^+^ Vero cells obtained by FISH-flow and the different viral inputs tested. Dotted red line indicates the amount of virus saturating the detection of SARS-CoV-2 RNA^+^/dsRNA^+^ Vero cells using this technique.

**
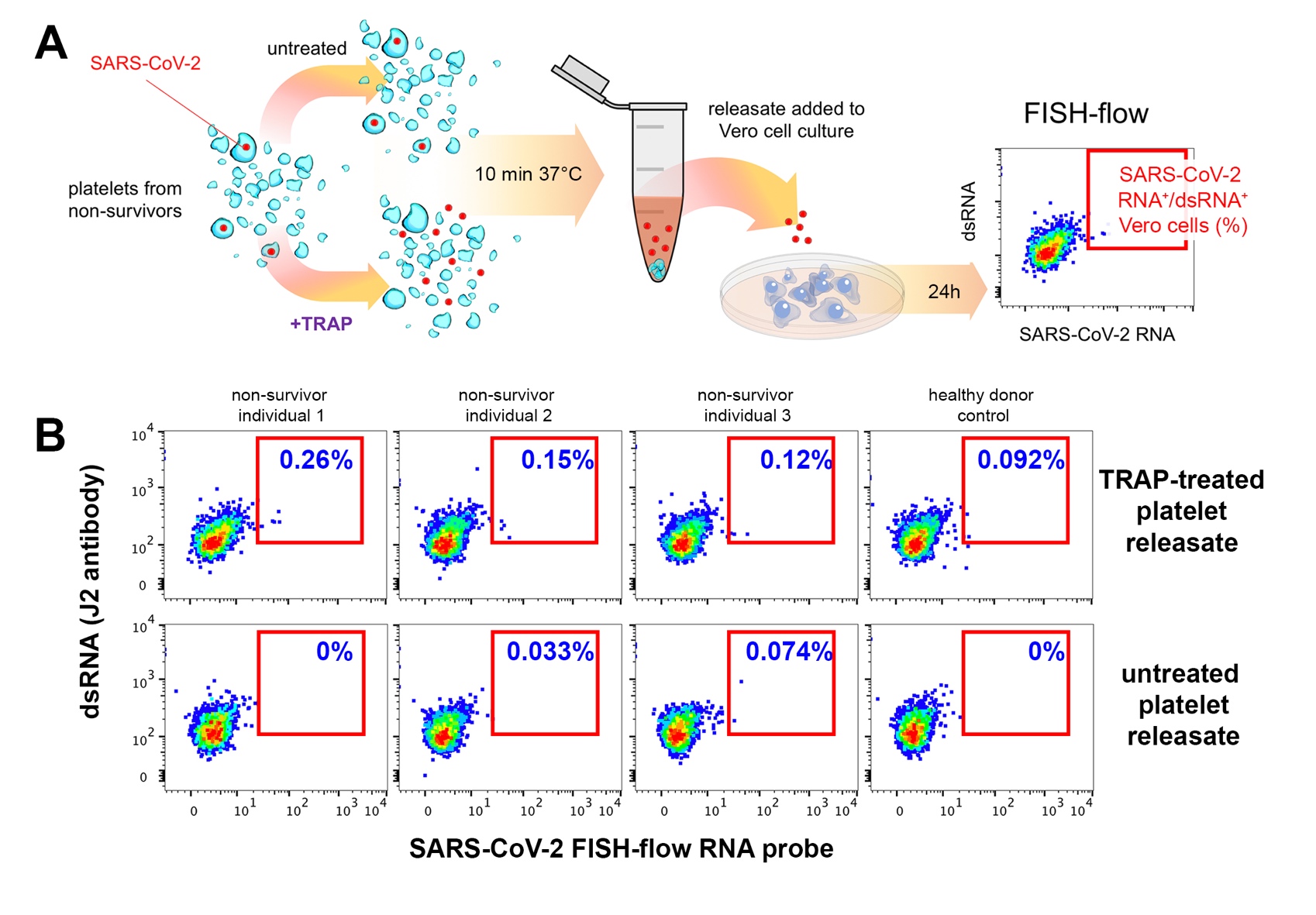
**

**Figure S9. Titration of SARS-CoV-2 within platelets using TRAP-treated releasates.**

(A) Scheme of the experiments using TRAP-activated platelet to purge SARS-CoV-2 from within platelets into platelet extracellular environment, referred to as releasate and titration of purged virus on Vero cells by FISH-flow for SARS-CoV-2 (+) RNA/ dsRNA.

(B) Percentage of FISH-flow SARS-CoV-2 RNA^+^/dsRNA^+^ Vero cells treated for 24 hours with releasates from platelets from non-survivor donor activated or not by TRAP (n=3 donors) and from healthy donor platelets as control. Gate in red shows the percentage of SARS-CoV-2 RNA^+^/dsRNA^+^ Vero cells among the entire Vero cell population.

**
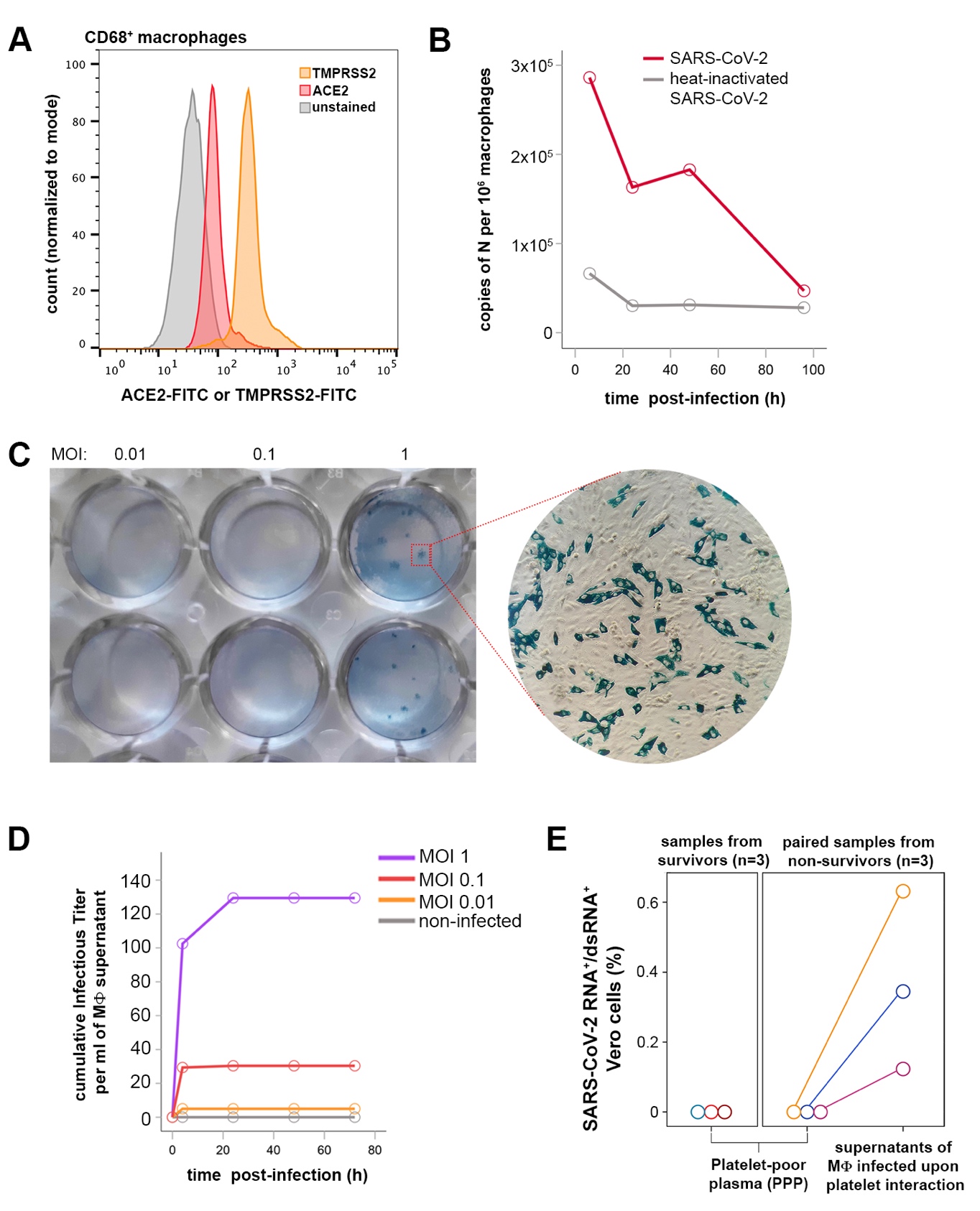
**

**Figure S10. Validation of macrophages as host cells for SARS-CoV-2 replication**

(A) Histogram showing the surface expression of ACE2 and TMPRSS2 by CD68^+^ macrophages gated in flow cytometry analysis.

(B) Productive infection of macrophages followed by 0, 6, 24, 48 and 96 hours post-infection with SARS-CoV-2 or heat-inactivated SARS-CoV-2 *in vitro*, as assessed by RT-qPCR of viral gene N in cell fractions of infected macrophage cultures. Data were normalized by the number of cells in culture as assessed by the expression of ACTB gene.

(C) Example of Focus Forming Assay (FFA) on Vero cells using supernatants of macrophages infected with SARS-CoV-2 at different multiplicities of infection (MOI) for 24 hours. SARS-CoV-2 foci on Vero cells are detected macroscopically (left) and microscopically (right).

(D) Cumulative Infectious Titers detected by FFA in supernatants of macrophages infected with SARS-CoV-2 at different MOI, collected at different time point post-infection.

(E) SARS-CoV-2 outgrowth (% SARS-CoV-2 RNA^+^/dsRNA^+^ Vero cells) obtained from tested supernatants of macrophages infected by non-survivor platelet samples and from PPP of the same non-survivor individuals pairwise (n=3 independent donors). PPP from survivors lacking virus in platelets were also tested as control (n=3 independent donors).

**Supplementary Tables**

**Table S1**: **Patient medications according to the hospital outcome (PRP samples)**

| **N (%)** | **Survivors, n=27** | **Non-survivors, n=25** | ***p*-value** |
| --- | --- | --- | --- |
| **COVID related medications** |  |  |  |
| Oxygen: |  |  |  |
| O2 nasal | 9 (33%) | 11 (44%) | 0.407 |
| O2 assisted ventilation | 9 (33%) | 11 (44%) | 0.407 |
| Immunomodulators: |  |  |  |
| Corticosteroids | 9 (33%) | 10 (40%) | 0.618 |
| Azythromycin | 16 (59%) | 9 (36%) | 0.096 |
| Tocilizumab | 0 (0%) | 2 (8%) | 0.261 |
| Others: Ivermectin | 2 (7%) | 1 (4%) | 0.604 |
| Others: NO | 1 (4%) | 1 (4%) | 0.956 |
| Hydroxychloroquine | 3 (11%) | 4 (16%) | 0.608 |
| Vasopressive | 6 (22%) | 12 (48%) | 0.055 |
| Antiplatelet | 3 (11%) | 1 (4%) | 0.356 |
| Anticoagulant | 16 (59%) | 13 (52%) | 0.599 |

**Table S2**: **Patient death causes**

| **N (%)** | **Non-survivors, n=25** |
| --- | --- |
| **Death causes** * |  |
| RF (+/- TA) | 15 (60%) |
| MOF | 4 (16%) |
| RF, MOF (+/- TA) | 3 (12%) |
| Encephalitis + RF | 1 (4%) |
| Pulmonary embolism | 1 (4%) |
| Unknown | 1 (4%) |

* Death causes: RF: respiratory failure; MOF: multiorgan failure, TA: therapeutic arrest.

**Table S3.** **List of FISH-flow RNA probe sequences**


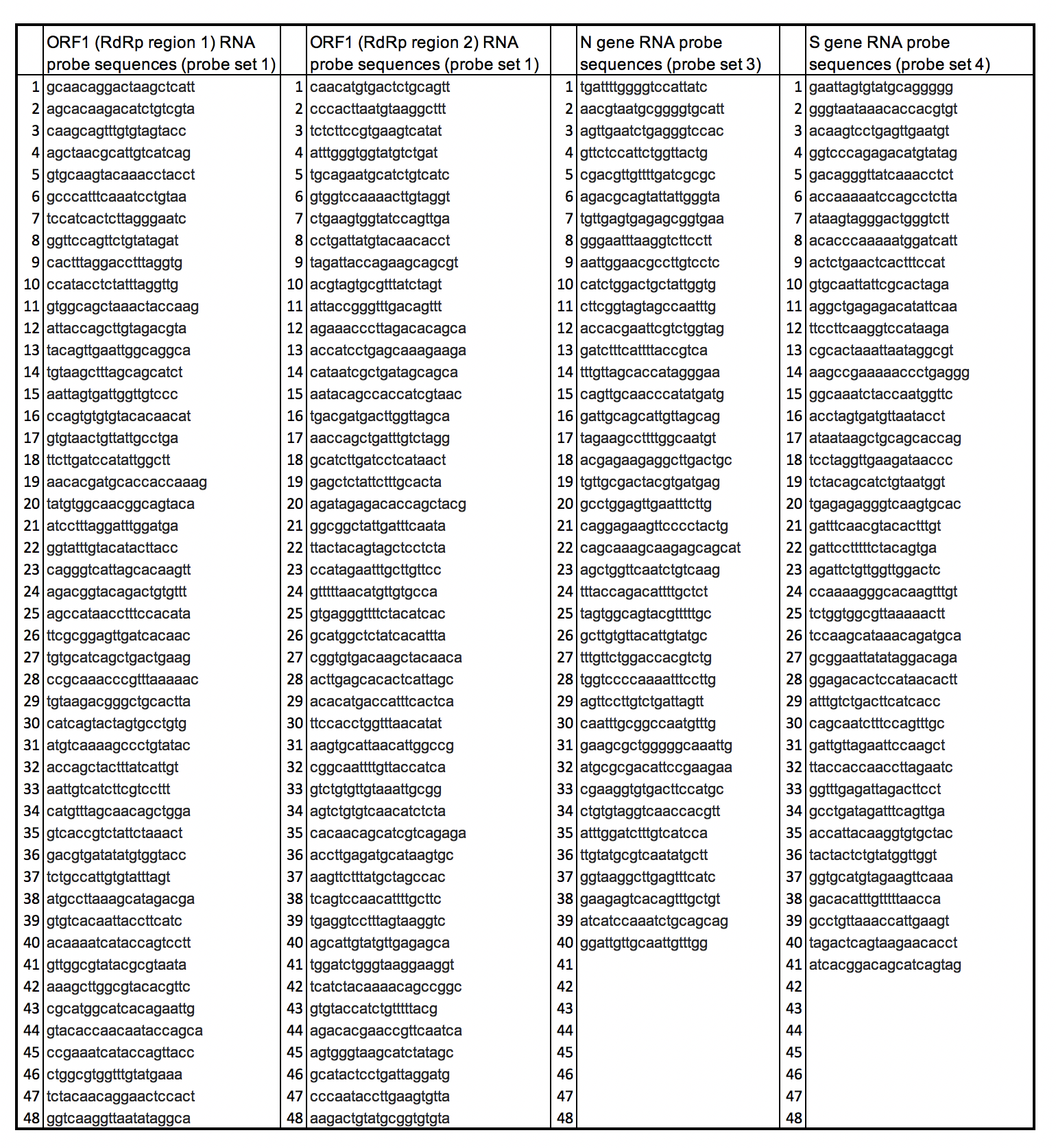

Supplement: Supplementary file 1 — Supplementary file1 (DOC 11555 KB) [file 18_2022_4318_MOESM1_ESM.doc]
